# Trafficking of High Avidity HER-2/neu-Specific T Cells into HER-2/neu-Expressing Tumors after Depletion of Effector/Memory-Like Regulatory T Cells

**DOI:** 10.1371/journal.pone.0031962

**Published:** 2012-02-16

**Authors:** Vivian L. Weiss, Timothy H. Lee, Hong Song, Theodore S. Kouo, Chelsea M. Black, George Sgouros, Elizabeth M. Jaffee, Todd D. Armstrong

**Affiliations:** 1 The Sidney Kimmel Cancer Center at Johns Hopkins, John Hopkins University School of Medicine, Baltimore, Maryland, United States of America; 2 Department of Oncology, John Hopkins University School of Medicine, Baltimore, Maryland, United States of America; 3 Graduate Program in Immunology, John Hopkins University School of Medicine, Baltimore, Maryland, United States of America; 4 Department of Radiology and Nuclear Medicine, John Hopkins University School of Medicine, Baltimore, Maryland, United States of America; 5 Graduate Program in Cellular and Molecular Medicine, John Hopkins University School of Medicine, Baltimore, Maryland, United States of America; 6 Graduate Program in Pharmacology, John Hopkins University School of Medicine, Baltimore, Maryland, United States of America; 7 The Skip Viragh Pancreatic Cancer Center, and Johns Hopkins University School of Medicine, Baltimore, Maryland, United States of America; MRC National Institute for Medical Research, United Kingdom

## Abstract

**Background:**

Cancer vaccines are designed to activate and enhance cancer-antigen-targeted T cells that are suppressed through multiple mechanisms of immune tolerance in cancer-bearing hosts. T regulatory cell (Treg) suppression of tumor-specific T cells is one barrier to effective immunization. A second mechanism is the deletion of high avidity tumor-specific T cells, which leaves a less effective low avidity tumor specific T cell repertoire available for activation by vaccines. Treg depleting agents including low dose cyclophosphamide (Cy) and antibodies that deplete CD25-expressing Tregs have been used with limited success to enhance the potency of tumor-specific vaccines. In addition, few studies have evaluated mechanisms that activate low avidity cancer antigen-specific T cells. Therefore, we developed high and low avidity HER-2/neu-specific TCR transgenic mouse colonies specific for the same HER-2/neu epitope to define the tolerance mechanisms that specifically affect high versus low avidity tumor-specific T cells.

**Methodology/Principal Findings:**

High and low avidity CD8^+^ T cell receptor (TCR) transgenic mice specific for the breast cancer antigen HER-2/neu (neu) were developed to provide a purified source of naïve, tumor-specific T cells that can be used to study tolerance mechanisms. Adoptive transfer studies into tolerant FVB/N-derived HER-2/*neu* transgenic (*neu*-N) mice demonstrated that high avidity, but not low avidity, neu-specific T cells are inhibited by Tregs as the dominant tolerizing mechanism. High avidity T cells persisted, produced IFNγ, trafficked into tumors, and lysed tumors after adoptive transfer into mice treated with a neu-specific vaccine and low dose Cy to deplete Tregs. Analysis of Treg subsets revealed a Cy-sensitive CD4^+^Foxp3^+^CD25^low^ tumor-seeking migratory phenotype, characteristic of effector/memory Tregs, and capable of high avidity T cell suppression.

**Conclusion/Significance:**

Depletion of CD25^low^ Tregs allows activation of tumor-clearing high avidity T cells. Thus, the development of agents that specifically deplete Treg subsets should translate into more effective immunotherapies while avoiding autoimmunity.

## Introduction

Activation, amplification, and survival of CD8^+^ cytotoxic T lymphocytes (CTL) are required as part of a successful adaptive immune response to developing tumors. High avidity antigen-targeted CTL can exit the thymus after selection, even though many recognize altered self-proteins. However, multiple mechanisms of peripheral immune tolerance exist that prevent complete activation of CTL, including ignorance, deletion, functional inactivation, and T regulatory cell (Treg)-mediated suppression [1], [2], [3], [4], [5], [6]. Low avidity CTL specific for the same antigenic epitope easily exit the thymus but are thought to remain non-functional in the periphery, presumably due to inefficient activation and maintenance of function. Due to efficient thymic deletion and peripheral suppression of high avidity T cells, low avidity T cells are the predominant population of T cells available for activation in the periphery of cancer bearing hosts. Therefore, model systems allowing tracking and the dissection of mechanisms of regulation of sub-populations of antigen-targeted CTL are needed to decipher barriers to CTL clearance of tumors in cancer bearing hosts.

Suppression by Tregs is one well-described tolerance mechanism. Tregs inhibit the activation and recruitment of antigen-targeted CTL. It is unclear whether Tregs suppress both high and low avidity antigen-targeted CTL, and how and where this suppression occurs in tumor-bearing hosts. Understanding the mechanism by which Tregs suppress these CTL is complicated by the recent discovery of Treg subsets that may be more potent suppressors of CTL, and may traffic more efficiently to areas of inflammation. Effector/memory-like CD4^+^Foxp3^+^ Tregs have been described that preferentially migrate to non-lymphoid and inflamed tissues to exert suppressive effects [7], [8]. These studies suggest that Tregs represent a heterogeneous population of cells responsible for a variety of suppressive and migratory functions. Therefore, it is critical to understand which Treg populations provide cancer-antigen-targeted CTL suppression within tumors so that agents can be identified to effectively suppress these sub-populations as part of immune activating therapies. The availability of naïve purified T cells with defined avidities that are specific for a cancer-targeted antigen allows for the dissection of the mechanisms involved in suppression, activation, and trafficking of antigen-targeted CTL in tolerant tumor-bearing hosts.

We previously demonstrated that FVB/N mice produce an oligoclonal high avidity T cell response against the immunodominant neu-derived CD8^+^ T cell epitope, RNEU_420–429_, when vaccinated with a neu and granulocyte-macrophage colony-stimulating factor (GM-CSF)-expressing whole cell vaccine. However, this same vaccine induces only low avidity RNEU_420–429_-specific T cells in *neu*-N mice that are naturally tolerant to neu due to the expression of the neu transgene in mammary tissue [9], [10]. We previously reported that 20–30% of *neu*-N mice treated with this vaccine and immune modulating doses of cyclophosphamide (Cy) to deplete Tregs develop high avidity RNEU_420–429_-specific CTL responses that are associated with tumor clearance. In contrast, mice developing only low avidity RNEU_420–429_-specific CTL fail to clear tumor [11]. These findings suggest that one difference in vaccine efficacy is the availability of high versus low avidity T cell repertoires for tumor clearance.

We previously generated high and low avidity RNEU_420–429_-specific CD8^+^ T cell clones derived from vaccinated FVB/N and *neu*-N mice, respectively [11]. While these clones are of value in studying antigen recognition, they are not ideal for studying mechanisms of tolerance because they have already been through the tolerance process. Here we report the generation of RNEU_420–429_-specific high and low avidity [12], [13] TCR transgenic mice that derive from these T cell clones. We show for the first time that tumor trafficking and function of the high avidity RNEU_420–429_-specific T cells when adoptively transferred into *neu*-N mice are suppressed by a CD25^low^ Treg effector/memory subpopulation residing at the tumor site. In addition, this suppression is reversed by depletion of Tregs with Cy. In contrast, low avidity T cells specific for the same antigenic epitope are not suppressed by this Treg sub-population and do not become activated following depletion of these Tregs. Thus, these findings elucidate one mechanism by which high avidity tumor antigen-specific T cells are selectively suppressed. These findings also provide a strong rationale for inhibiting specific subsets of Tregs as a component of cancer-targeting vaccine approaches.

## Results

### High avidity RNEU_420–429_-specific T cells mediate tumor regression only following Treg depletion in tolerant *neu*-N mice

**Figure 1:**
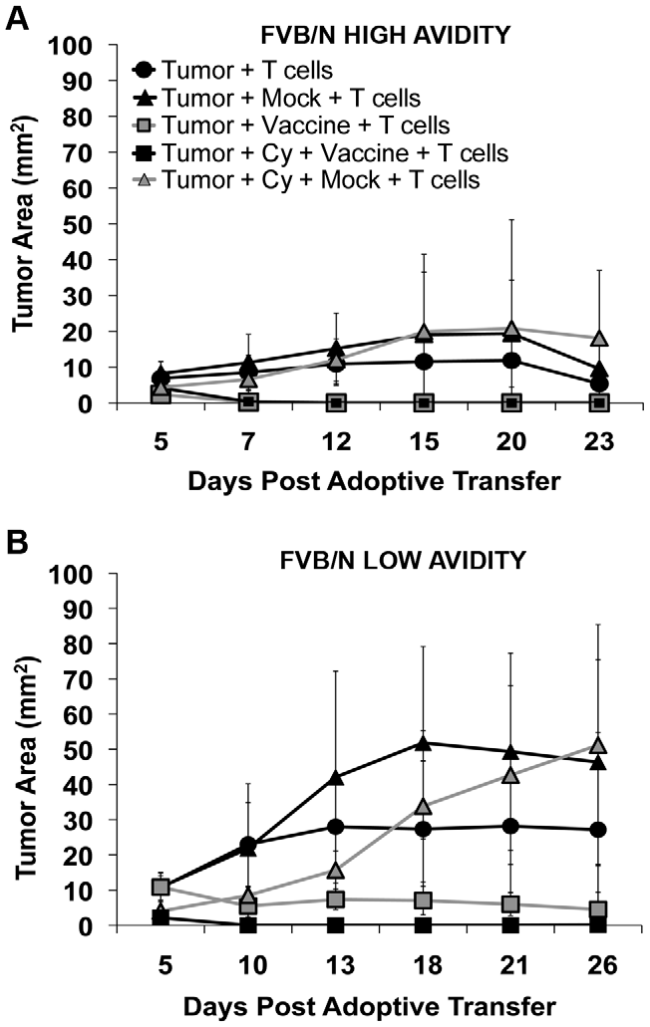
Adoptively transferred RNEU_420–429_-specific high avidity T cells enhance tumor clearance in FVB/N mice. The minimal tumorigenic dose was given to FVB/N mice on day 0; Cy was given on day 2; vaccine or mock vaccine was injected on day 3; high avidity or low avidity T cells were adoptively transferred on day 4. Treatment groups are indicated in the figures. (**A**) Tumor growth after high avidity T cell adoptive transfer into FVB/N mice. (**B**) Tumor growth after low avidity T cell adoptive transfer into FVB/N mice. Data was graphed as the tumor area vs. days (figures 1 A–B) after adoptive transfer. * = p<0.05; ** = p<0.001. Experiments were repeated three times (n = 10 mice per group).

We previously reported that Cy given one day prior to a neu-targeted vaccine resulted in the partial depletion of Tregs, activation of endogenous high avidity RNEU_420–429_–specific CD8^+^ T cells, and tumor clearance in 20–30% of *neu*-N mice [11]. However, it is unclear whether the failure to cure the majority of tumor bearing mice was due to the inefficient depletion of Tregs, lack of induction of sufficient numbers of high avidity T cells, or additional mechanisms of T cell regulation. RNEU_420–429_-specific high and low avidity CD8^+^ TCR transgenic mice were therefore created to evaluate the fate of naïve antigen-targeted T cells under conditions of T cell tolerance [11]. These mice were confirmed to express high and low avidity T cell receptors specific for the RNEU_420–429_ epitope (as previously reported for the T cell clones from which they derived) using dilutional tetramer staining of naïve TCR transgenic T cells isolated directly from the TCR transgenic mice (**Figure S1**), and using ELISpot as a functional analysis of avidity. Specifically, comparison of high versus low avidity T cell populations isolated from vaccinated TCR transgenic mice and assessed either directly or following a one week stimulation with the RNEU_420–429_ peptide, demonstrated that the high avidity T cell populations were better cytokine producers when tested against NT2.5 tumors as targets (Data not shown). To assess the *in vivo* function of each T cell population, isolated naïve high and low avidity T cells were adoptively transferred into tumor-bearing FVB/N and tolerant *neu*-N mice, treated with combinations of either the specific 3T3neuGM vaccine or the negative control 3T3GM mock vaccine, with or without low dose Cy. 3T3GM mock vaccine was used as the negative control in all studies in order to control for the non-specific physiological effects of GM-CSF. Hence any differences between groups are from the specific presence of neu in the vaccine. FVB/N mice were used as positive control mice to assess optimal T cell function in non-tolerant mice. FVB/N mice receiving high avidity T cells and the 3T3neuGM vaccine with or without Cy cleared tumor faster than mice receiving low avidity T cells (**Figure 1a, b**) indicating that the high avidity T cells are naturally more potent than the low avidity T cells even in mice without tolerizing mechanisms. In addition, treatment with the high avidity T cells and only the control 3T3GM mock vaccine still controlled tumors in the FVB/N mice more effectively than treatment with low avidity T cells and the 3T3GM mock vaccine (**Figure 1a, b**) indicating that high avidity T cells can be activated in non-tolerant mice bearing antigen expressing tumors even without receiving an antigen containing vaccine.

**Figure 2:**
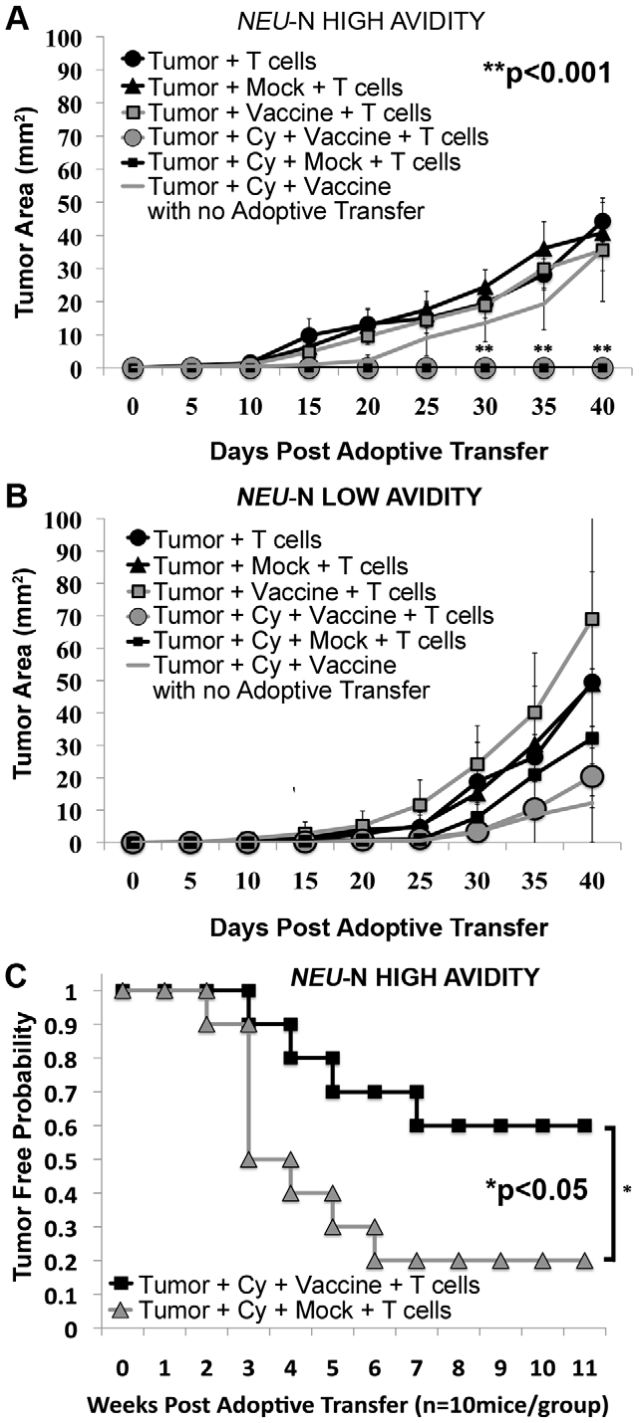
RNEU_420–429_-specific high avidity T cells enhance tumor clearance in *neu*-N mice given Cy and vaccine. *Neu*-N mice were treated as in Figure 1 and the Methods. (**A**) Tumor growth after high avidity T cell adoptive transfer into *neu*-N mice. (**B**) Tumor growth after low avidity T cell adoptive transfer into *neu*-N mice. (**C**) Twice the minimum tumor dose was given to *neu*-N mice (1×10^5^ cells/mouse) on day 0. Data was graphed as the tumor free probability vs. weeks (Figure 2C) or tumor size (mm^2^) vs. days (figures 2A–B) after adoptive transfer. * = p<0.05; ** = p<0.001. Experiments were repeated three times (n = 10 mice per group).

The majority of *neu*-N mice (>75%) receiving Cy plus high avidity T cells also cleared tumor rapidly, within one week of adoptive transfer. After 30 days, tumors in these mice were significantly smaller in size than tumors in mice treated with Cy plus vaccine, without high avidity T cells (**Figure 2a**, p<0.001 using both ANOVA and t test). In contrast, tumors in *neu*-N mice receiving low avidity T cells all progressed, regardless of the treatment they received (**Figure 2b**). In addition, the rate of tumor growth in mice receiving Cy, 3T3neuGM vaccine, and low avidity T cells, was similar to mice receiving Cy and vaccine alone. These data indicate that Cy is required for the sustained *in vivo* function of high avidity but not low avidity T cells in tolerant mice. However, these studies failed to show a difference in tumor clearance between mice receiving the 3T3neuGM vaccine versus the 3T3GM mock vaccine when given with both Cy and high avidity T cells in FVB/N and *neu*-N mice. Therefore, to further investigate whether a neu-targeted vaccine is needed for optimal high avidity T cell function when Tregs are depleted, we compared the mock versus the neu-targeted vaccine for the ability to facilitate clearance of a larger tumor burden. When mice were challenged with twice the number of tumor cells, a significantly greater percentage of mice receiving the neu-targeted vaccine had long-term tumor control (p<0.05, **Figure 2c**). Similar results were seen if T cell transfer was delayed until 10 days post tumor challenge allowing the tumors to develop longer (Data not shown). Using titration experiments, we found that minimal number of adoptively transferred T cells to eliminate large tumor burdens was 4×10^6^ T cells. (Data not shown) Thus, both Treg depletion and an antigen-specific vaccine are required for inducing the most effective high avidity T cell response against larger tumor burdens in these tolerant mice.

### Adoptively transferred high avidity T cells produce higher levels of cytokines when compared with low avidity T cells in FVB/N and *neu*-N mice

**Figure 3:**
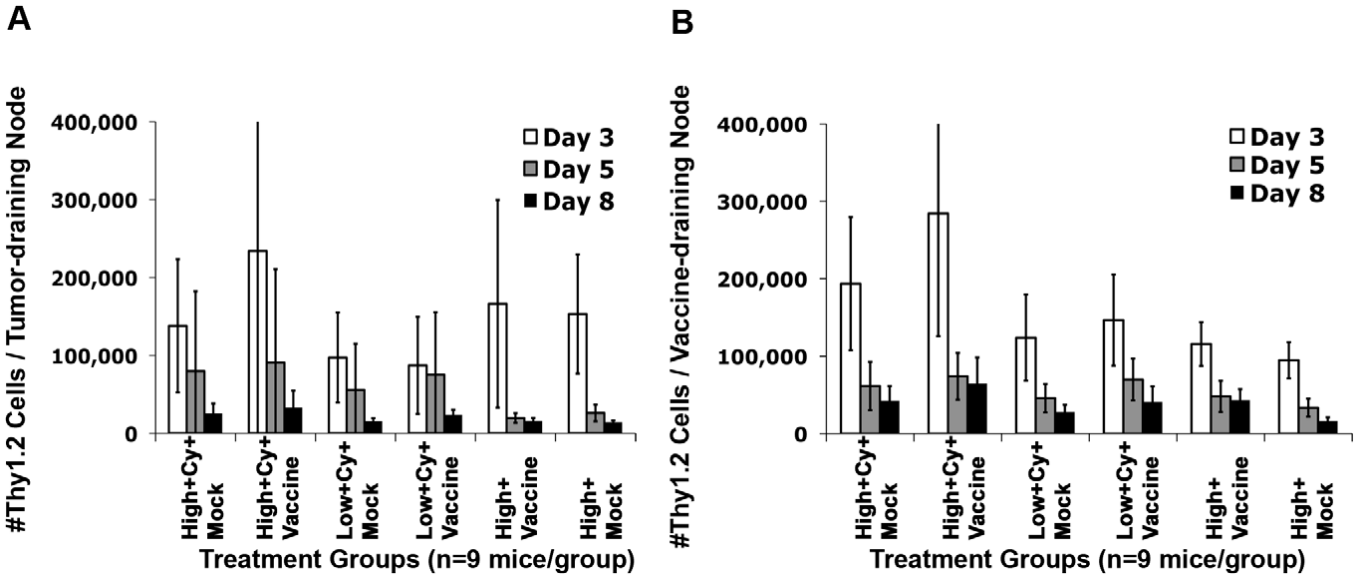
High avidity T cells expand preferentially after adoptive transfer into Cy and vaccine-treated *neu*-N mice. Mice were injected with 1×10^6^ tumor cells/mouse one week prior to Cy and vaccine treatment. High avidity or low avidity T cells were adoptively transferred one day after vaccination. (**A**) The absolute # of Thy1.2 T cells were calculated in the tumor draining lymph nodes (TDNs) of *neu*-N mice on day 3 (White bars), 5 (Gray bars), and 8 (Black bars) after adoptive transfer (n = 9 mice per group). (**B**) The absolute # of Thy1.2 T cells were calculated in the vaccine draining lymph nodes (VDNs) on day 3 (White bars), 5 (Gray bars), and 8 (Black bars) after adoptive transfer (n = 9 mice per group). Treatment groups are as indicated. High/low = High or low avidity T cells, respectively. Cy = Cytoxan treated. Vaccine = 3T3neuGM vaccine, Mock = 3T3GM control vaccine. Each experiment was repeated three times with similar results.

Initial adoptive transfer studies demonstrated that high avidity T cells are more capable of killing neu-expressing tumor cells than low avidity T cells. To assess whether this difference correlated with increased high avidity T cell persistence, we evaluated the percentage of high avidity and low avidity T cells (TCR transgenic T cells are Thy1.2^+^ and *neu*-N and FVB/N mice are Thy1.1^+^) recovered from FVB/N and *neu*-N mice at 1, 3, and 5 weeks after adoptive transfer. While high avidity T cells persisted for at least 5 weeks following transfer into tumor-bearing FVB/N mice treated with the 3T3neuGM vaccine plus Cy (**Figure S2a**, p<0.001), less than 2% of both high avidity and low avidity T cells persisted >3 weeks in *neu*-N mice even following treatment with 3T3neuGM vaccine plus Cy (**Figure S2b**). We previously reported that maximal Treg depletion occurs 2 days after Cy treatment in *neu*-N mice and returns to baseline about one week later [11]. Therefore, the effects of Treg depletion on T cell persistence would be expected to occur during T cell priming in the first week after vaccine plus Cy treatment. We therefore calculated the absolute numbers of T cells remaining in the tumor-draining lymph nodes (TDN) and vaccine-draining lymph nodes (VDN) of *neu*-N mice on days 3, 5, and 8 after adoptive transfer. Both high avidity and low avidity T cells were found to migrate into the VDN and TDN, regardless of the treatment (**Figure 3a, b**). In addition, the highest numbers of T cells were detected in VDN and TDN on day 3 after adoptive transfer, a finding consistent with previous studies suggesting that the peak of endogenous T cell priming occurs in the lymph nodes 3–4 days following treatment with the 3T3neuGM vaccine plus Cy [**14**]. Thus, although there were a higher number of high versus low avidity T cells detected in TDN and VDN, differences in T cell persistence do not fully explain why high but not low avidity T cells develop tumor lytic function following Treg depletion.

**Figure 4:**
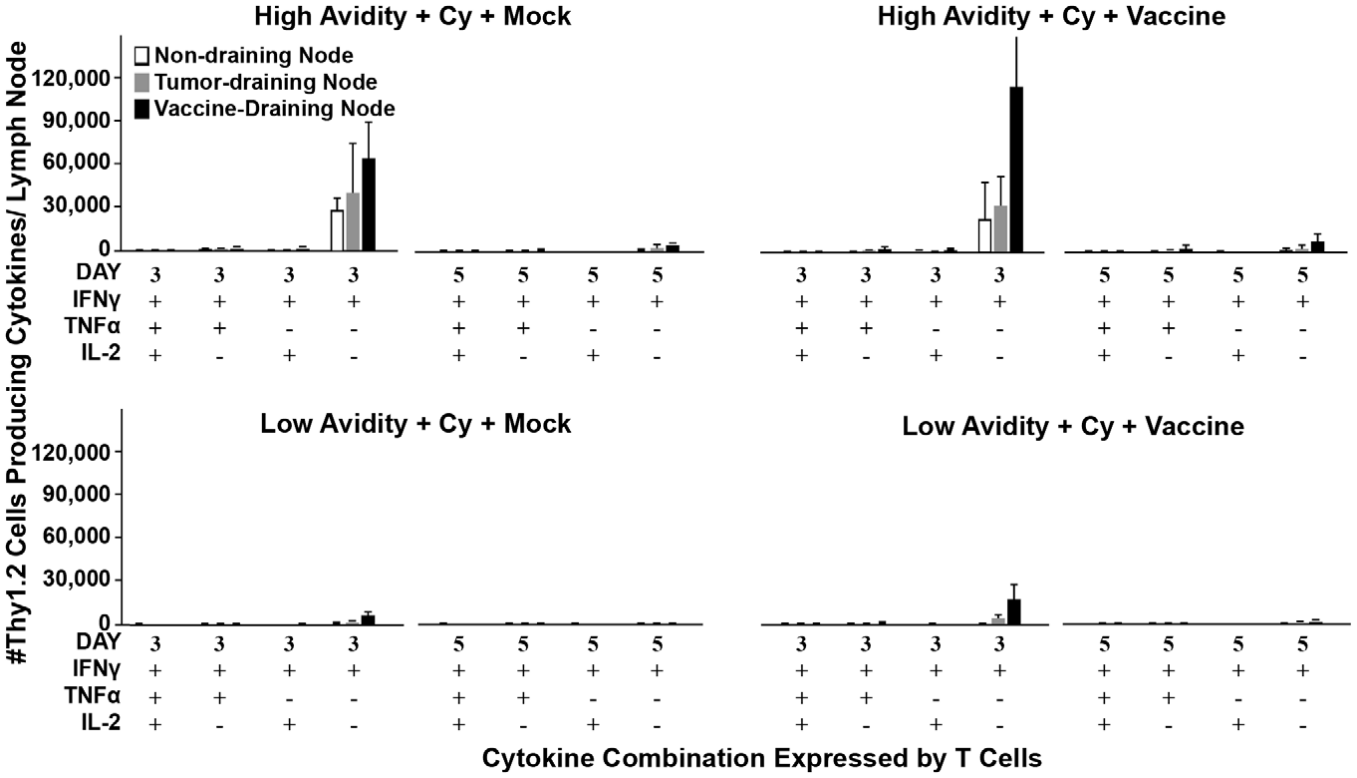
Enhanced cytokine secretion in high avidity T cells post-transfer into Cy and vaccine-treated *neu*-N mice. On day 3 and day 5 after adoptive transfer, lymphocytes isolated from the non-draining lymph nodes (NDNs, White bars), VDNs (Black bars), and TDNs (Gray bars) were analyzed for the ability to secrete IFNγ, TNFα, and IL-2 secretion by ICS, and the absolute # of activated Thy1.2 cells was calculated (n = 3 mice per group). Treatment groups are as indicated. High/low = High or low avidity T cells, respectively. Cy = Cytoxan treated. Vaccine = 3T3neuGM vaccine, Mock = 3T3GM control vaccine. Each experiment was repeated three times with similar results.

We therefore evaluated the activation status of the high avidity and low avidity T cells in Cy plus 3T3neuGM-treated *neu*-N mice on days 3 and 5 after adoptive transfer. Specifically, the absolute number of IFNγ-, TNFα-, and IL-2-secreting Thy1.2^+^ cells in the VDN, TDN, and non-draining lymph nodes (NDN, control lymph nodes) were determined. Despite the persistence of similar numbers of total high and low avidity adoptively transferred T cells, there were significantly greater numbers of high avidity T cells secreting activation-associated cytokines when compared to low avidity T cells (**Figure 4**). IFNγ is the main cytokine produced by these activated high avidity T cells in tolerant *neu*-N mice, while multiple cytokines (IFNγ, TNFα, and IL-2) are produced following adoptive transfer of high avidity T cells into non-tolerant mice (**Figure S3**). Taken together, these findings suggest that the tolerant environment in *neu*-N mice hinders the development of the most potent polycytokine producing T cells, even when Tregs are transiently suppressed during T cell priming. However, high but not low avidity T cells transferred into tolerant mice are able to reach a level of activation required to produce IFNγ and mediate tumor lysis.

### Adoptively transferred high avidity but not low avidity T cells upregulate integrin and CXCR3 expression in TDNs in vaccinated and Cy treated *neu*-N mice

**Figure 5:**
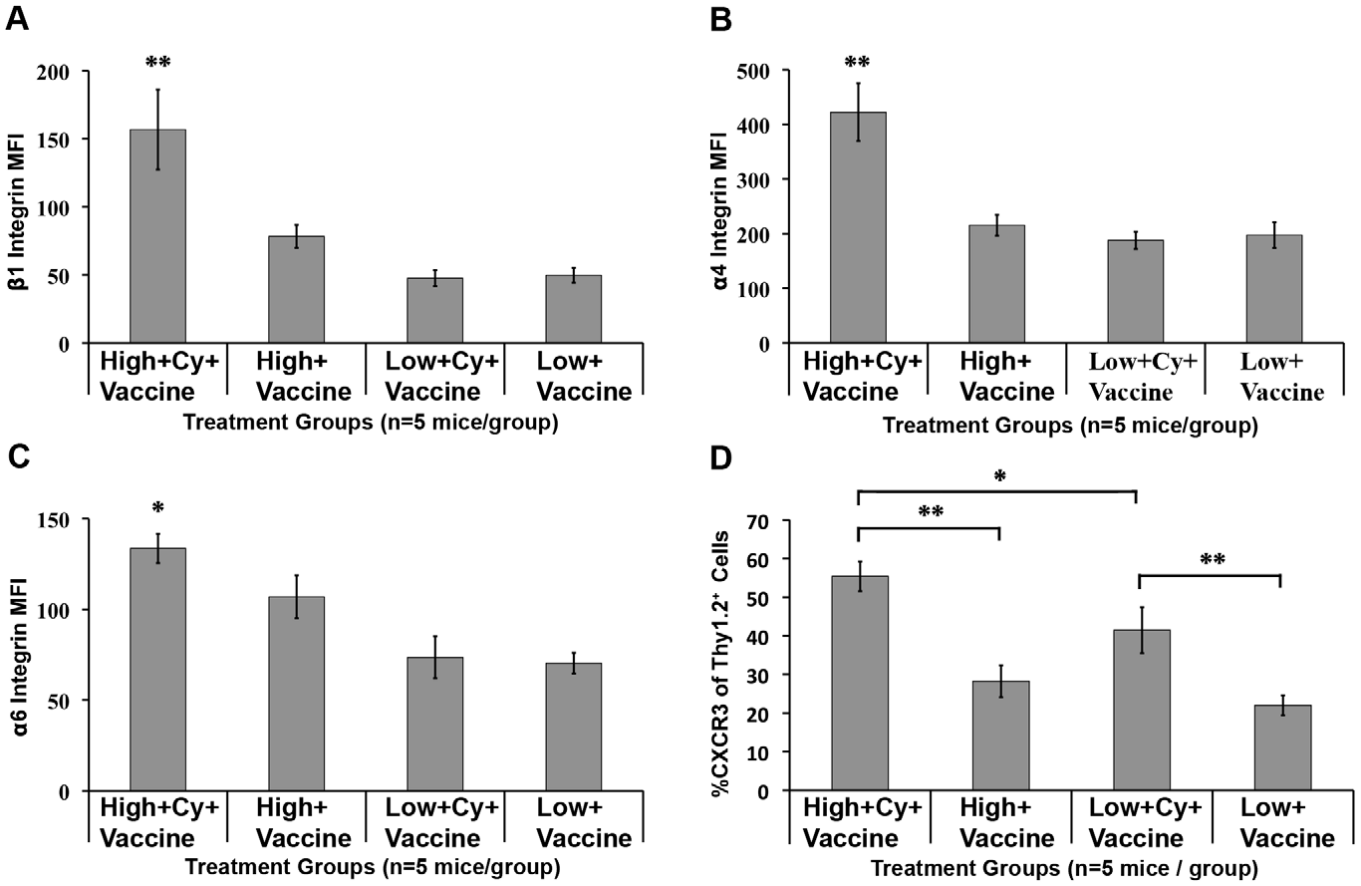
Cy plus vaccine enhances VLA-4, VLA-6, and CXCR3 expression on high avidity T cells. On day 3, TDN lymphocytes from *neu*-N mice were stained for (**A**) β1 integrin expression, (**B**) α4 integrin, or (**C**) α6 integrin, and the Mean Fluorescent Intensities (MFI) calculated. (n = 3mice/group). Isotype control MFIs were β1 integrin ∼20, α4 integrin ∼50–100, α6 integrin ∼30. (**D**) TDN lymphocytes from *neu*-N mice on day 5 were stained for CXCR3 and the %CXCR3^+^ of Thy1.2 cells was calculated (n = 5 mice/group). MFI isotype for CXCR3 is ∼10. * = p<0.05, ** = p<0.001. High/low = High or low avidity T cell transfer, respectively. Cy = Cyclophosphamide treatment. Vaccine = 3T3neuGM vaccine, Mock = 3T3GM control vaccine. Each experiment was repeated three times with similar results.

So far the data demonstrate that adoptively transferred high avidity T cells can be activated to secrete cytokines to a greater extent than low avidity T cells in 3T3neuGM plus Cy-treated *neu*-N mice. However, activated T cells must also migrate from the lymph nodes to the tumor microenvironment to effect tumor clearance. It is still unclear whether there are also differences in tumor migration efficiency between high and low avidity T cells in *neu*-N mice. T cell expression of integrins and chemokines are known requirements for T cell migration from the priming site to the site of effector function [15], [16]. Specifically, the VLA family of molecules are known mediators of T cell activation and migration in models of tumor and inflammation [17], [18], [19], [20], [21]. We therefore compared high and low avidity T cells for surface expression of the VLA integrins β1 (CD29), α4 (CD49d), and α6 (CD49f), in spleens and TDNs, on days 3 and 5 after adoptive transfer into tumor-bearing *neu*-N mice. High avidity T cells in TDNs had significantly higher β1 integrin expression when compared with low avidity T cells isolated from 3T3neuGM plus Cy-treated (p<0.001) and 3T3neuGM only treated mice (p<0.001 for both ANOVA and t test) (**Figure 5a**). High avidity T cells from 3T3neuGM plus Cy-treated mice also expressed significantly higher α4 (p<0.001 for both ANOVA and t test) and α6 (p<0.001 for ANOVA analysis and p<0.05 for t test) integrins than all other mouse groups (**Figure 5b, c**). Similar results were observed for T cells isolated from spleen (data not shown). Thus, Cy treatment leads to upregulation of T cell trafficking integrins on high avidity but not low avidity T cells.

Chemokine receptor CXCR3 expression has also been reported to be critical for T cell migration to target tissues expressing the CXCR3 ligands CXCL9 and CXCL10. CXCL9 is produced in the tumor-microenvironment in response to IFNγ production, and can attract CXCR3^+^ T cells into the tumor [22], [23]. To further evaluate differences between high and low avidity T cell function in tolerized mice, we analyzed CXCR3 expression on both high avidity and low avidity T cells on days 3 and 5 after adoptive transfer into tumor-bearing *neu*-N mice. High avidity T cells isolated from the TDNs upregulated surface expression of CXCR3 to a greater extent than low avidity T cells from 3T3neuGM plus Cy-treated mice at day 5 (p<0.05, **Figure 5d**). In contrast, high avidity T cells from 3T3neuGM only treated mice had lower levels of CXCR3 expression (p<0.001). In addition, we analyzed CXCR3 and CXCL9 expression in neu-expressing tumors on days 3 and 5 after adoptive transfer into tumor-bearing *neu*-N mice. Interestingly, while tumors isolated from all mouse groups produced low levels of CXCL9, *neu*-expressing tumors isolated from mice receiving the 3T3neuGM vaccine, Cy, and high avidity T cells, produced the highest levels of CXCL9 on days 3 and 5 after adoptive transfer (**Figure S4**). These data suggest that CXCR3 expression is upregulated on high avidity T cells following Cy treatment, allowing them to migrate to the CXCL9-producing tumor. These data provide an additional mechanism by which Tregs may preferentially regulate high avidity but not low avidity T cells in tolerant mice.

### Cy-mediated Treg depletion facilitates high avidity T cell infiltration into *neu*-N mouse tumors

**Figure 6:**
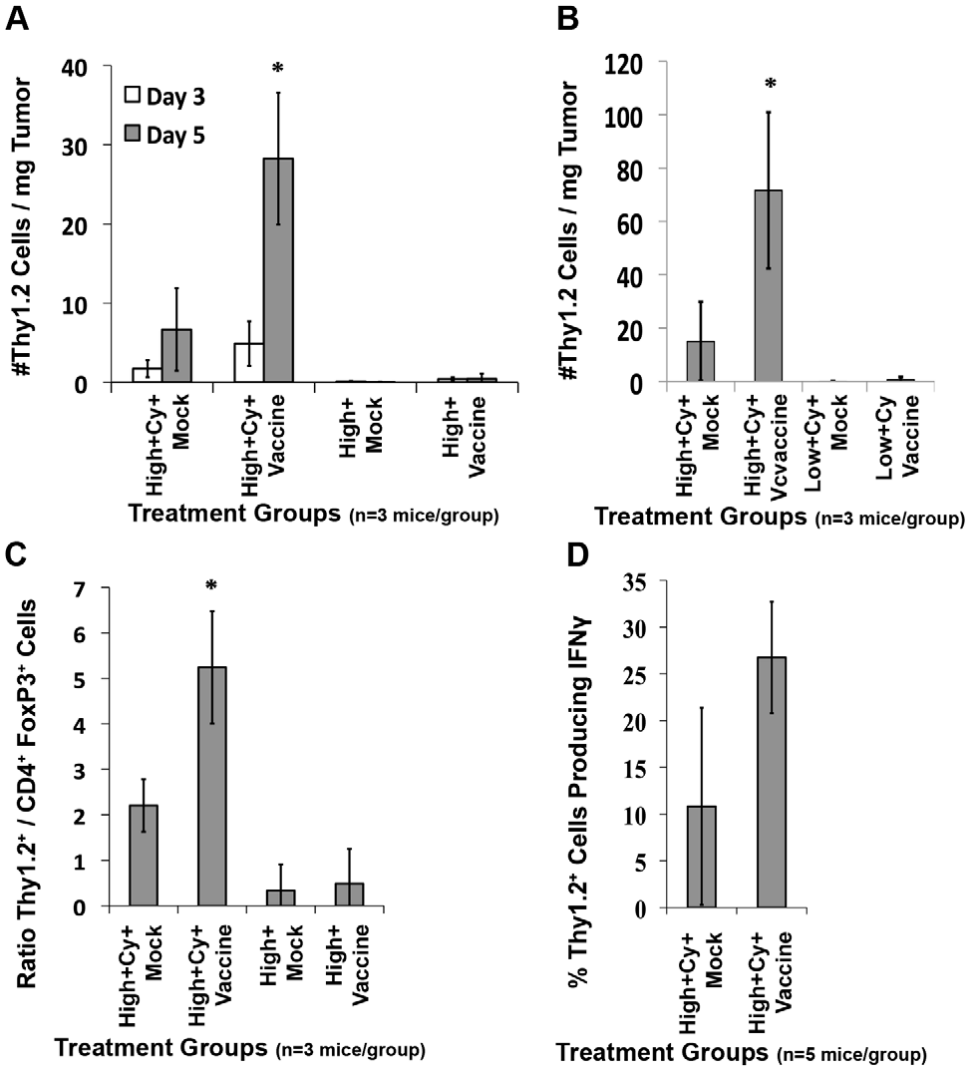
Cy-mediated Treg depletion increases high avidity T cell tumor trafficking in *neu*-N mice. *Neu*-N mice were treated as in **Figure 3**. (**A and B**) On day 3 (White bars) and day 5 (Gray bars) after adoptive transfer, the absolute number of Thy1.2^+^ cells/mg of tumor was calculated for each treatment group (n = 3 mice/group). (**C**) On day 5 after adoptive transfer the ratio of Thy1.2^+^ cells/CD4^+^Foxp3^+^ cells was calculated (n = 3 mice/group). (**D**) Lymphocytes from tumors on day 5 after adoptive transfer were stimulated as described in the methods and the %Thy1.2^+^ cells producing IFNγ after RNEU_420–429_ stimulation was calculated after NP stimulation was subtracted (n = 5mice/group). * = p<0.05. High/low = High or low avidity T cell transfer, respectively. Cy = Cyclophosphamide treatment. Vaccine = 3T3neuGM vaccine, Mock = 3T3GM control vaccine. Each experiment was repeated three times with similar results.

The adoptive transfer studies presented thus far demonstrate that Cy given in sequence with an antigen-targeted vaccine to deplete Tregs selectively increases high but not low avidity T cell persistence, activation, and integrin expression in VDNs and TDNs. To further evaluate Cy-mediated high avidity T cell function, we investigated the numbers and quality of high avidity T cells migrating into the tumor after various treatments. High avidity and low avidity T cells were adoptively transferred into tumor-bearing FVB/N and *neu*-N mice receiving either the 3T3neuGM or control 3T3GM mock vaccines. Significantly greater numbers of high avidity T cells were isolated from the tumors on day 5 after adoptive transfer following vaccine plus Cy treatment when compared with all other treatment groups (p<0.05 using ANOVA and t test, **Figure 6a**) and few low avidity T cells infiltrated tumors regardless of the treatment (**Figure 6b**). Interestingly, the peak of high avidity T cell infiltration into tumors occurs about 2 days after the peak of high avidity T cell IFNγ secretion and integrin upregulation in TDNs. As expected, both low avidity and high avidity T cells infiltrated the tumors of vaccinated FVB/N mice, even without Treg depletion (data not shown). Thus Cy-mediated Treg depletion is required for high avidity but not low avidity T cell activation and tumor migration in tolerized mice. Low avidity T cell migration likely requires additional activation signals that are not available in non-tolerant hosts.

While CD8^+^ T cell trafficking into the tumor is an important predictor of tumor clearance, the ratio of effector T cells to Tregs (Teff/Treg) and the activation status of effector T cells in the tumor microenvironment are also important tumor clearance factors [24], [25], [26], [27], [28]. We therefore compared the number of tumor infiltrating high avidity T cells to the number of CD4^+^Foxp3^+^ Tregs in the tumor of vaccinated mice with and without Cy treatment (**Figure 6c**). Cy treatment groups did indeed have a higher Teff/Treg ratio in the tumor when compared to non-Cy treated groups (p<0.05 using ANOVA and t test). To confirm that these high avidity T cells in TIL are capable of effector function, ICS studies were performed and demonstrated that up to 30% of the high avidity TIL produce IFNγ (**Figure 6d**). These data confirm through *ex-vivo* analysis, that high avidity T cells trafficking into the tumors are present in high effector∶Treg ratios, and are capable of producing IFNγ.

**Figure 7:**
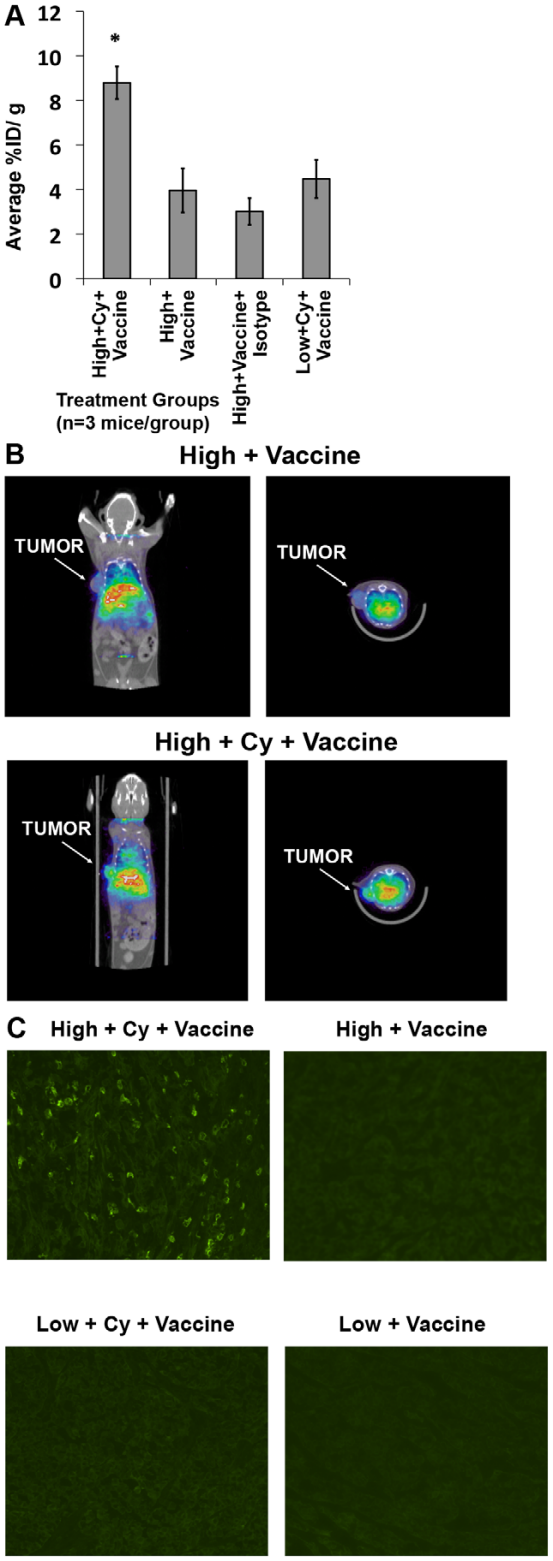
Confirmation of increased high avidity T cell tumor trafficking by *in vivo* imaging. (**A**) *Neu*-N mice were tumor challenged and treated as in the Methods. One day after T cell adoptive transfer mice were treated with In-111 labeled anti-Thy1.2, the tumors isolated and the radiation content analyzed as described in Methods. Isotype = Isotype control treatment group. Tumor radioactivity concentration was reported as the average % injection dose/g of tumor (n = 3mice/group). (**B**) *neu*-N mice treated as in **7A**, and underwent SPECT/CT imaging. Coronal (left panel) and transverse (right panel) cross-sections through the tumor are shown for each treatment. (**C**) Tumors were collected on day 5 after adoptive transfer and immunofluorescence stained for Thy 1.2. * = p<0.05. High/low = High or low avidity T cell transfer, respectively. Cy = Cyclophosphamide treatment. Vaccine = 3T3neuGM vaccine. Mock = 3T3GM Control Vaccine. These experiments were repeated 2–3 times with similar results.

Next, we used biodistribution and imaging studies to verify the selective trafficking of high avidity versus low avidity T cells into the tumors of tolerant *neu*-N mice. Tumor-bearing *neu*-N mice received the 3T3neuGM or 3T3GM mock vaccines with and without Cy, and high or low avidity T cells, followed by injection with indium-111-labeled Thy1.2 mAb on day 4 after T cell transfer. Twenty-four hours following Thy1.2 monoclonal antibody injection, tumors were excised and measured for indium-111 content. The mean tumor radioactivity concentration was 2-fold higher in mice receiving 3T3neuGM vaccine plus Cy with high avidity T cells than in the non-Cy groups (p<0.05 using ANOVA and t test) (**Figure 7a**). These findings were further confirmed using SPECT/CT imaging, *in vivo* (**Figure 7b**). Finally, we performed *in situ* immunofluorescence staining of resected tumors for Thy1.2 expression (**Figure 7c**). From these studies it is clear that only Thy1.2^+^ high avidity T cells infiltrate tumors predominantly following Cy treatment. These results confirm that high avidity T cells have the capacity to infiltrate tumors of tolerant mice if the mice are given immune-modulating doses of Cy in combination with vaccine.

### CD25^low^ Tregs migrate into tumors and mediate high avidity T cell suppression

**Figure 8:**
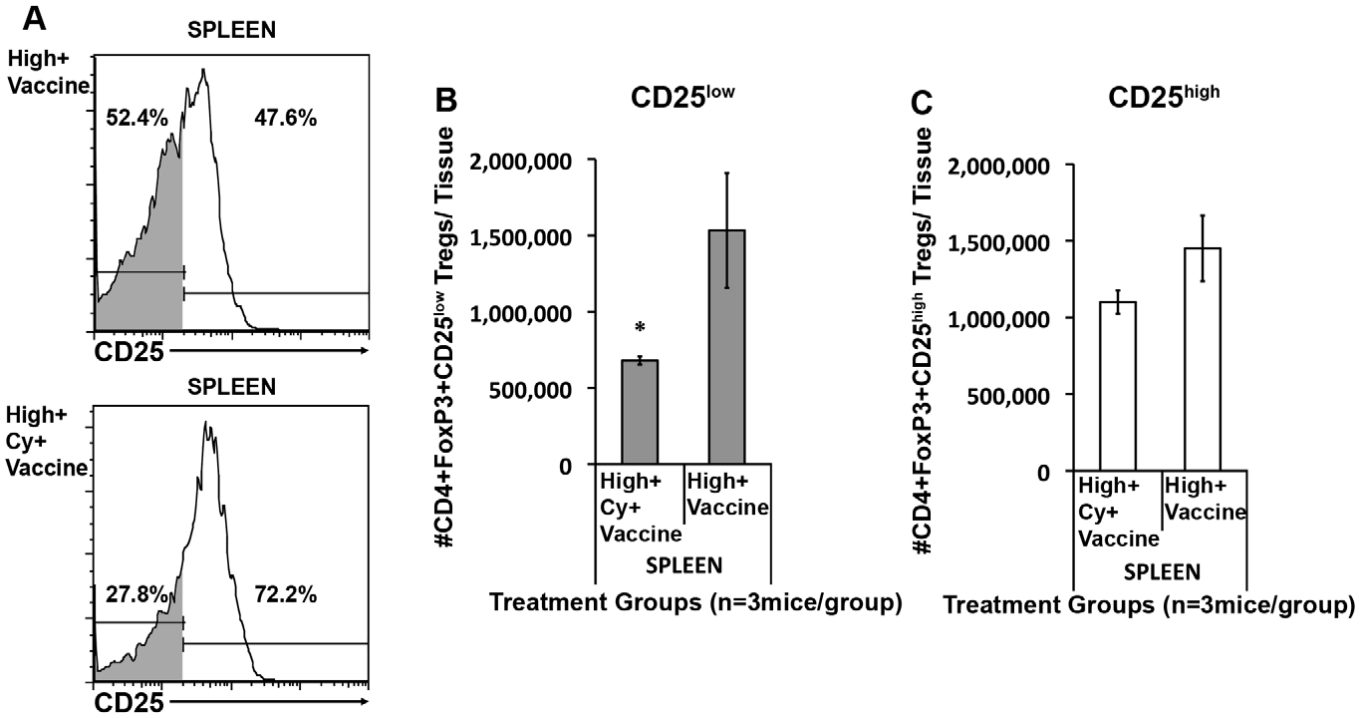
Cy preferentially depletes CD25^low^Foxp3^+^ Tregs. *Neu*-N mice were treated as in **Figure 3** and the Methods. Lymphocytes from spleens were collected on day 3. (**A**) CD4^+^Foxp3^+^ Tregs were analyzed for CD25 expression. Definition of CD25^high^ vs. CD25^low^ is based on isotype control. CD25^low^ cells = gray fill under the histogram. (**B**) Absolute # CD25^low^CD4^+^Foxp3^+^ Tregs calculated in spleens. (**C**) Absolute number of CD25^high^CD4^+^Foxp3^+^ Tregs calculated in spleens. High = high avidity T cell transfer, Cy = Cyclophosphamide treatment, Vaccine = 3T3neuGM vaccine treatment. * = p<0.05. These studies were repeated three times with similar results (n = 3 mice/group).

While Cy allows for enhanced high avidity T cell trafficking into tumors, it does not deplete the systemic Treg population as extensively as other Treg-depleting agents. One explanation for this difference is that each Treg inhibiting agent affects different Treg subsets. Recent studies have characterized distinct Treg subpopulations that express different activation molecules, migration integrins, and levels of the IL-2 receptor, CD25. Initial flow cytometry experiments characterizing the CD4^+^Foxp3^+^ T cells that infiltrate neu-expressing tumors showed that Cy depletes a population of CD4^+^Foxp3^+^ T cells that express an activated/migratory phenotype (ICOS^+^, β-integrin^high^, CD44^+/high^, CD62L^low^) (Data not shown), Subsequently this subset of Tregs was further analyzed for CD25 expression following treatment of *neu*-N mice with 3T3neuGM vaccine alone or 3T3neuGM plus Cy and high avidity T cells. Cy given with the 3T3neuGM vaccine reduced both the percent and absolute number of CD25^low^ Tregs in the spleen compared to vaccine alone (28% versus 52% of the CD4^+^Foxp3^+^ cells were CD25^low^, respectively) (**Figure 8a, b**). The reduction in absolute numbers of CD25^low^ Tregs with Cy treatment in spleen reached significance (p<0.05 and p<0.001, respectively using both ANOVA and t test, **Figure 8b, c**). Trends were similar but not statistically significant in TDNs (Data not shown). These data provide additional support that Cy preferentially targets a CD25^low^ Treg population.

**Figure 9:**
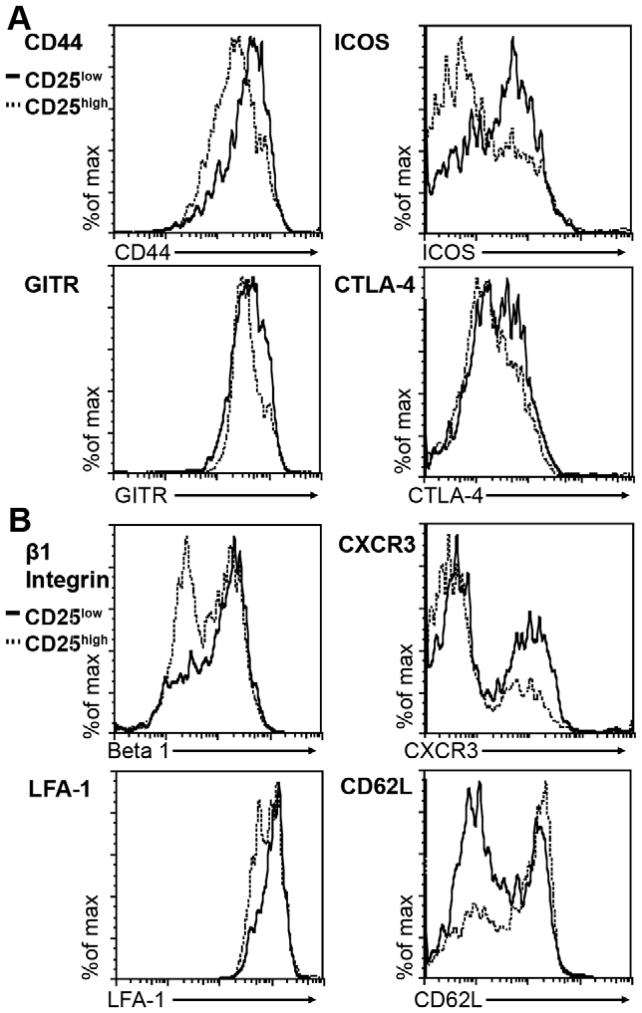
CD25^low^Foxp3^+^ Tregs demonstrate an effector phenotype. (**A**) CD4^+^Foxp3^+^CD25^high^ and CD4^+^Foxp3^+^CD25^low^ Tregs from high avidity T cell and vaccine treated mice analyzed for expression of ICOS, CTLA-4, GITR, and CD44. (**B**) CD25^high^ and ^low^ Tregs were analyzed for β1 integrin, LFA-1, CD62L, and CXCR3. CD25^high^ histograms = Black line. CD25^low^ histograms = Dotted line. These studies were repeated three times with similar results (n = 3 mice/group).

CD25^low^ Tregs have been described as an inflammation-seeking, migratory effector/memory population responsible for suppression in the periphery, while CD25^high^ Tregs have been described as a lymph-node resident population [7], [8]. To further assess the migration potential and activation status of these two Treg subsets, Tregs from *neu*-N mice treated with vaccine and high avidity T cells were isolated and analyzed based on CD25 expression. Analysis of each population revealed that the CD4^+^Foxp3^+^CD25^low^ Tregs expressed higher levels of the activation markers ICOS, CD44, CTLA-4, and GITR, and the migration markers β1 integrin, LFA1, and CXCR3 and lower levels of the lymph node homing marker CD62L when compared with the CD4^+^Foxp3^+^CD25^high^ Tregs (**Figure 9a, b**). Thus, CD25^low^ Tregs express an activated phenotype [7], [8] that appears to be responsible for suppressing high avidity T cells infiltrating tumors.

**Figure 10:**
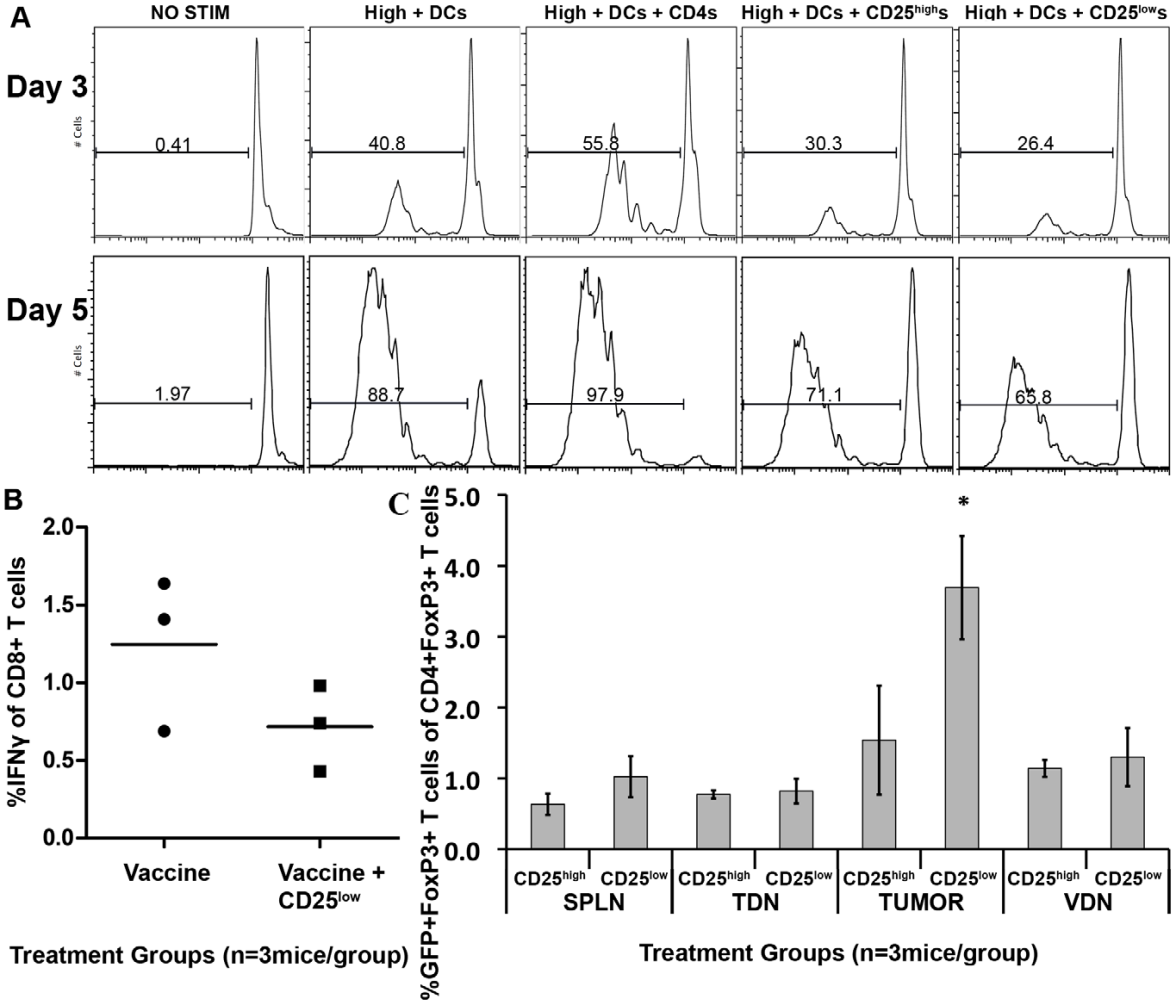
CD25^low^Foxp3^+^ Tregs suppress high avidity T cells and traffic preferentially to the tumor. (**A**) CD25^low^ and CD25^high^ CD4^+^ Treg cells suppress high avidity T cell activation *in vitro*. CD25^low^ or CD25^high^ CD4^+^ FoxP3^gfp+^, DC, and CFSE-labeled high avidity Thy1.2^+^ CD8^+^ neu-specific T cells were mixed as described in the Methods. CFSE dilution was measured on days 3 and 5. CD4^+^ T cells from FVB/N mice were used as controls. High+DCs = High avidity T cells+DCs. High+DCs+CD4s = High avidity T cells+DCs+FVB/N CD4^+^ T cells, (**B**) CD25^low^CD4^+^FoxP3^+^ Tregs trend toward suppression *in vivo*. *neu*-N/FoxP3^gfp^ mice were given tumor and vaccinated as in the Methods. CD4^+^CD25^low^ Tregs were isolated 1 week later and transferred to FVB/N-TgN(TIE2GFP)287Sato mice which were then vaccinated. CD8^+^ T cells were isolated 2 weeks later and tested for the ability to secrete IFNγ in response to RNEU_420–429_. Data are presented as % RNEU_420–429_-specific, IFNγ^+^ of CD8^+^ T cells. This experiment was done twice with similar results. (**C**) CD25^low^ Tregs traffic preferentially to the tumor. *neu*-N mice received tumor and one week later received Cy, vaccine, 6×10^6^ high avidity T cells, and 5×10^5^ CD25^low^ or CD25^high^ CD4^+^GFP^+^ Tregs. On day 5 CD25^low^ or CD25^high^ CD4^+^GFP^+^ Tregs recovered from tumors, spleens, TDNs, and VDNs were enumerated as a fraction of the total # of FoxP3^+^ T cells at each site (n = 3mice/group). * = p<0.05. This experiment was repeated three times with similar results.

Next, we assessed whether CD4^+^Foxp3^+^CD25^low^ Tregs can suppress antigen-specific high avidity T cells using an *in vitro* suppression assay. Naïve CFSE-labeled high avidity T cells were incubated with CD11c^+^ DCs from 3T3neu GM vaccine-treated FVB/N mice for 3 and 5 days to stimulate their proliferation. These vaccine primed DCs from non-tolerized mice would be expected to provide optimal high avidity T cell activation. Flow cytometry sorted CD4^+^Foxp3^+^CD25^high^ or CD4^+^Foxp3^+^CD25^low^ Tregs isolated from *Foxp3^GFP^ neu*-N mice that were vaccinated with 3T3neuGM one week earlier were then added to the high avidity T cell cultures. Treatment of *neu*-N mice with vaccine alone would be expected to induce optimal numbers of Tregs. High avidity T cells were analyzed for CFSE dilution after 3 and 5 days of culture. Positive controls included high avidity T cells with DCs alone, and high avidity T cells with DCs plus control naïve CD4^+^ T cells isolated from FVB/N GFP mice. As expected, the addition of FVB/N CD4^+^ T cells increased the proliferation of high avidity T cells above that seen with DCs and high avidity T cells alone. However, high avidity T cell proliferation was suppressed at 3 and 5 days in the groups that received either CD25^high^ or CD25^low^ Tregs, indicating that CD25^low^ Tregs are indeed capable of suppressing tumor-specific high avidity T cell proliferation (**Figure 10a**). In support of these findings, both Treg subsets were confirmed to express TGFβ and IL-10 by qRT-PCR (data not shown). Similarly, these same CD4^+^Foxp3^+^CD25^low^ Tregs derived from vaccinated *Foxp3^GFP^ neu*-N mice suppressed high avidity T cell IFNγ secretion *in vivo* after they were transferred into tumor bearing FVB/N mice treated with the 3T3neuGM vaccine and high avidity T cells (**Figure 10b**). To further establish that the CD25^low^ Tregs are the predominant Tregs that traffic into tumors, we evaluated the percent of Tregs found in tumors after each Treg subpopulation was adoptively transferred into tumor bearing mice. *Neu*-N mice were given 3T3neuGM vaccine plus Cy, and adoptively transferred high avidity T cells. CD25^low^ CD4^+^Foxp3^+^ or CD25^high^ CD4^+^Foxp3^+^ Tregs (5×10^5^ per mouse) from vaccinated *Foxp3^GFP^ neu*-N mice were also adoptively transferred on the same day as the high avidity T cells. Five days later, the percentage of transferred GFP^+^ Treg subsets within the total CD4^+^Foxp3^+^ population was evaluated in the spleens, VDNs, TDNs, and tumors. Although both Treg subsets traffic into the lymph nodes and spleen, the CD25^low^ Tregs traffic to a significantly greater extent into the tumor than the CD25^high^ Tregs (p<0.05 using both ANOVA and t test) (**Figure 10c**). These data provide additional support that the CD25^low^ Treg population is primarily responsible for high avidity T cell suppression in the tumor microenvironment.

## Discussion

We previously reported that high avidity CD8^+^ T cell populations specific for the immunodominant neu-derived epitope, RNEU_420–429_, are recruited to kill neu-expressing tumors following treatment with a neu-expressing vaccine and low dose Cy as a method to deplete Tregs. Here we report three new findings that for the first time elucidate the mechanism by which Tregs suppress high avidity T cell activation and migration in the immune tolerant environment of *neu*-N mice. First, high avidity cancer antigen-targeted T cells can effectively eradicate developing tumors in tolerant hosts when efficient numbers of activated T cells are available. However, activation and persistence of efficient numbers of high avidity T cells is only possible following reduction of Treg numbers. Importantly, we show for the first time that Tregs appear to be the major regulator of these high avidity effector T cells. Second, we also show for the first time that antigen-targeted low avidity T cells are unable to persist long-term and reach a necessary threshold of activation in tolerant hosts, even with Treg reduction. Third, a Cy-sensitive Foxp3^+^CD25^low^ effector/memory Treg sub-population present in tolerant mice is the primary mediator of antigen-targeted high avidity T cell suppression within the tumor microenvironment.

Ideal vaccines should induce antigen-specific high avidity T cells that can migrate to sites of inflammation and express multiple effector cytokines [4], [29], [30], [31]. In practice, vaccines do induce high avidity T cells specific for infectious and cancer-associated antigens when administered prior to antigen exposure. However, there is an ongoing debate in the literature as to whether cancer antigen-specific high avidity T cells exist in the periphery of cancer-bearing hosts because many of these antigens are altered self proteins. Some studies have suggested that high avidity T cells are deleted, leaving only low avidity T cells for vaccine activation [32]. Other studies have shown that high avidity antigen-targeted T cells exist, but are tolerized and unable to mediate tumor clearance [6], [33], [34]. We previously reported that Cy mediated Treg depletion given in sequence with a neu-targeted vaccine induces endogenous high avidity T cells that mediate tumor clearance in 20–30% of mice [11]. These prior studies could not address whether the low number of treatment successes was due to the existence of yet undefined tolerance mechanisms or the inability to induce large enough numbers of antigen-specific high avidity T cells. Using the transgenic T cells described here to model endogenous T cells, we demonstrate that larger numbers of vaccine-activated high avidity T cells are necessary to treat developing tumors in tolerized mice. Furthermore, these data suggest that vaccination alone is not enough to activate the number of endogenous T cells required for tumor clearance, and that a significant reduction in the numbers of a specific subset of Tregs is required to allow efficient vaccine activation of high avidity T cells. Thus, these data show that Tregs are indeed a main mechanism by which high avidity tumor antigen-specific T cell function is suppressed.

In addition to inducing high avidity T cells, an ideal vaccine should also induce a sufficient memory response to fight off future antigenic challenges [35], [36]. Our studies also show that approximately 60% of mice given Cy, vaccine, and adoptive transfer of high avidity T cells can resist a second tumor challenge given 3 weeks post adoptive transfer, indicating that our vaccine regimen can induce a partial memory type response. At this time point, Thy1.2^+^ high avidity T cells comprise a detectable population of the CD8^+^ T cells with effector and central memory phenotypes in *neu*-N mice that wanes at 5 weeks (Figure S2, and Data not shown). The partial response may be due to the re-establishment of tolerance in this system either by the re-establishment of Treg numbers, as we have shown, or by an as yet undetermined mechanism [11]. Future experiments will address the longevity of the high avidity response in this system. However, we hesitate to make definitive conclusions regarding the memory phenotype of our adoptively transferred T cells, as others have clearly demonstrated that adoptively transferring high numbers of T cells alters the effector memory/central memory development ratio away from the endogenous situation [37], [38], [39].

Partial Treg depletion using low dose Cy plus a neu-targeted vaccine allowed for enhanced persistence, activation, and tumor-trafficking of adoptively transferred high avidity T cells, leading to tumor clearance in tolerant *neu*-N mice. Thus, the availability of sufficient high avidity T cells alone is likely not enough to treat tumors in tolerant hosts. Instead, Cy treatment, either through Treg reduction or another mechanism, facilitates efficient high avidity T cell activation, persistence, and tumor trafficking. Studies have shown that lymph node residing T cells upregulate VLA-4, VLA-6, and CXCR3 in order to migrate to CXCL9 producing tumors [15], [18], [20]. In our model, high avidity T cells transferred into Cy-treated *neu*-N mice upregulated these trafficking receptors after adoptive transfer, allowing for tumor-infiltration, and tumor clearance by one week after Cy administration, which is the same time-point when depleted Treg populations begin to repopulate the tolerant host [11], and the transferred high avidity T cells lose IFNγ production. Thus, Cy-mediated Treg depletion allows high avidity T cells to upregulate the migration and activation signals necessary for tumor trafficking and anti-tumor activity. Until methods are available to deplete Tregs for longer than 1–2 weeks, it is not possible to determine whether vaccine induced endogenous T cells are capable of reaching the numbers achieved with adoptively transferred high avidity T cells that are required for tumor control in the majority of mice.

Although adoptively transferred high avidity T cells are efficiently activated to clear tumor in tolerant mice, their cytokine secretion capacity appears limited to IFNγ secretion. In contrast, these same high avidity T cells produce multiple cytokines (IFNγ, IL-2, and TNFα) when transferred into tumor-bearing non-tolerant FVB/N mice. Other groups have suggested that TNFα expression correlates with better effector function, cell persistence [40], and clinical outcomes [41]. Thus, these data imply that a reduction in Treg numbers alone is not sufficient to optimally activate high avidity T cells. It is likely that fewer numbers of activated high avidity T cells would be required to clear the same tumor burden in tolerant *neu*-N mice if these T cells were optimally activated. In support of this concept, the activation state of the high avidity T cells has recently been implicated in their ability to promote tumor clearance. Specifically, central memory T cells have been described as more “fit” to mediate tumor clearance due to their less-differentiated state and higher proliferative capacity [42], [43], [44]. Activation of terminally-differentiated high avidity T cells is thought to lead to weaker responses due to their limited proliferation. However, high avidity T cells, including central memory cells, are often exhausted due to chronic tumor antigen stimulation and may exist in a “corrupted” or tolerant state [44]. Thus, additional studies are needed to further clarify the role that the T cell activation state plays in tumor clearance in tolerant hosts.

Many studies have also focused on antigen-targeted low avidity T cell activation and function due to the predominance of low avidity T cells in cancer-bearing hosts. Previous studies suggested that low avidity T cells remain ignorant of antigen expression and therefore do not mount a successful tumor-specific response [32], [45], [46], while others suggest that CD4^+^ T cell help enhances low avidity T cell function allowing for some degree of tumor destruction [26], [46]. While those studies attempted to address the role of low avidity T cells in tumor-bearing mice, none had available a source of naïve low avidity CD8^+^ TCR transgenic T cells specific for a natural tumor antigen. Here, we show that neu-specific low avidity T cells are capable of activation when adoptively transferred into a non-tolerant environment. In *neu*-N mice, however, low avidity T cells were unable to reach a level of activation necessary for adequate IFNγ secretion, integrin upregulation, or tumor infiltration. As a result, these low avidity cells did not affect tumor clearance, and their activation and migration status was not altered by Treg reduction. The difference in low avidity T cell function in the non-tolerant versus tolerant environment is likely due in part to the presence of effective CD4^+^ T cell help in non-tolerant mice. Our finding agrees with other studies that show that with sufficient CD4^+^ T cell help, low avidity CD8^+^ T cells can become active and infiltrate the tumor microenvironment [47], [48]. Since low avidity T cells were able to migrate into the tumor, but still were not as potent as the high avidity T cells in rejecting tumor in non-tolerant FVB/N mice, it is likely that alternative mechanisms are blocking optimal low avidity T cell activation even in a more immune permissive setting.

Both CD25^high^ and ^low^ Treg subsets effectively suppress high avidity T cells *in vitro*, yet we only observed a trend in suppression with an *in vivo* suppression assay. One possibility that explains why the *in vivo* suppression assay results failed to reach significance is the technical difficulty of isolating sufficient numbers of Tregs that maintain stable function after adoptive transfer. It is also possible that systemic evaluation of CD25^low^ Treg suppression of high avidity T cells is not an optimal assay since CD25^low^ Tregs likely function best within the tumor micro-environment, where they preferentially migrate [49]. Some studies have shown that tumor-specific T cells are tolerized at the tumor site. Although technically difficult, future *in vivo* studies will attempt to address where CD4^+^Foxp3^+^CD25^low^ Tregs affect tolerance.

Emerging data demonstrate that even among CD4^+^FoxP3^+^ Tregs, there are sub-populations with different functions and sites of activity. To date, there are only a few studies that have attempted to characterize an inflammation-seeking and highly suppressive CD25^low^ Treg effector/effector memory population [7], [8], [50], [51]. The surface markers expressed by these Tregs point towards their critical suppressive role in tumor-bearing hosts. High β1 integrin, LFA-1, and CXCR3 expression on CD25^low^ Tregs allow for increased blood vessel extravasation and migration into the tumor, while the low levels of CD62L indicate a migratory phenotype. The importance of ICOS expression by Tregs has been well documented and may be responsible for increased IL-10 production [52], [53], while CD44, CTLA-4, and GITR are also expressed highly on antigen experienced or activated Tregs [54]. Although a few studies have shown that this CD25^low^ suppressor T cell population also express CD103 [7], we were unable to detect any Treg CD103 expression on CD25^low^ Tregs in *neu*-N mice. Other groups have describe Tbet expression by CD25^low^ Treg cells [8]. The CD25^low^ Tregs in our model also express high levels of CXCR3 and Tbet. (Data not shown) Taken together, these findings suggest that the CD25^low^ Tregs identified in this study likely represent an effector/memory Treg subpopulation similar to that described by other groups.

Treg depletion is believed to be an important strategy in enhancing cancer vaccine efficacy in tumor-bearing hosts. Many pre-clinical and clinical studies have successfully used a variety of Treg-depleting agents to reduce tolerance and activate CD8^+^ T cell populations in the setting of tumor development [55], [56]. Treg depletion strategies strive to deplete all Treg populations because we do not understand which populations predominantly prevent T cell activation in tumor-bearing hosts. Therefore, the study of Treg subpopulations may allow the development of new Treg depleting agents that remove the Tregs responsible for inhibiting cancer antigen-targeted T cells and preserve Treg populations required for inhibiting autoimmune disease. Thus, the surface activation markers expressed on the subset of Tregs that migrate to the tumor may be important targets for future Treg depletion strategies to improve vaccine therapy. This study also verifies the importance of this CD25^low^ Treg population in a clinically relevant model of breast cancer.

In addition to its CD25^low^ Treg-depletion effects, Cy may also have other beneficial effects in a tumor-bearing host. A few studies have shown that in certain models, Cy increases type I IFN production and activates DCs that increase effector cell populations [57], [58]. These findings may explain the apparent longer-term effects of Cy treatment on the persistence and activation of adoptively transferred high avidity T cells into FVB/N mice when compared with the short-term effects on the same T cells adoptively transferred into *neu*-N mice. It is possible that Cy depletes tolerogenic DCs or other antigen presenting cells responsible for activating Tregs [57], [59]. The resulting shift in DC homeostasis would ultimately lead to increased activation of tumor-specific CD8^+^ T cells. This effect would likely be short-term in *neu*-N mice due to the regeneration of such antigen presenting cell populations, whereas the effect would be longer-term in non-tolerant FVB/N mice. In addition, by increasing type I IFNs and DC activation, Cy may increase T cell survival and memory development [58], [60]. Recently there has been a link postulated between lymphopenia and autoimmune disease. Since Cy does cause mild lymphopenia, in the *neu*-N system, a mild form of autoimmunity may be induced leading to tumor destruction [61]. Finally, a recent study suggested that Cy may promote the differentiation of non-Treg CD4^+^ T cells into pro-inflammatory T helper 17 cells that enhance anti-tumor immunity [62]. Therefore, Cy may have multiple tolerance-reducing effects that lead to enhanced anti-tumor activity.

In conclusion, these findings demonstrate for the first time that high avidity antigen-targeted CD8^+^ T cells are regulated predominantly by Tregs in cancer bearing hosts. In addition, a subset of effector/memory Tregs are largely responsible for suppressing high avidity T cells in the tumor microenvironment. Low avidity tumor-specific T cells can function in a non-tolerant host following antigen-targeted vaccination but require as of yet undefined activation signals to achieve adequate function in tolerant hosts and optimal function in non-tolerant hosts. Thus, through the use of high avidity and low avidity T cell populations specific for the same antigen, the specific requirements for T cell activation, trafficking, and function within the tumor micro-environment can be delineated under different tumor-bearing conditions. Ultimately, this information will lead to improved combinatorial immune based approaches for cancer therapy.

## Methods

### Ethics Statement

All mice were treated according to AALAC approved protocols and guidelines outlined by the Johns Hopkins School of Medicine's Animal Care and Use Committee, specifically approved under protocol number MO10M262.

### Mice

All mice used were females between 6–12 weeks of age. FVB/N mice were obtained from Harlan Laboratories (Frederick, MD). *Neu*-N mice were purchased from the Jackson Laboratory (Bar Harbor, ME) and bred in the Johns Hopkins animal facility. FVB/N Thy1.2^+^ mice were generated by backcrossing the Thy1.2 gene from BALB/c mice onto the FVB/N background for 10 generations.

TCR transgenic mice were created from high avidity and low avidity RNEU_420–429_-specific T-cell clones, which were generated from vaccinated FVB/N and *neu*-N mice, respectively [11]. The high avidity clone expresses the TCR variable regions Vβ4 and Vα1.1, while the low avidity clone expresses the TCR variable regions Vβ2 and Vα5. cDNA was isolated from the clones using the SMART-RACE kit (Clontech) and primers HT-27 (TCGGTGAACAGGCAGAGGGTG) and HT-2 8(CTTTTGATGGCTCAAACAAGGAGA), which are specific for the alpha and beta constant regions of the TCR, respectively. Chains were cloned into pCR2.1 using the TOPO-TA cloning kit (Invitrogen). Artificial introns and restriction sites for cloning were constructed for the alpha and beta chains. The constructs were then cloned into cassette vectors as described by Kouskoff et al. [63], [64]. Low avidity α and β chains were PCR amplified from the genomic sequence and cloned directly into the Kouskoff cassette vectors [64].

Founder mice were selected following antibody staining for Vβ4 (clone KT4) and Vβ2 (clone B20.6) chains for the high avidity and low avidity mice, respectively (**Figure S1a–d**). Since antibodies to the Vα1.1 and Vα5 chains are not commercially available, the Vα chain usage was confirmed through evaluation of mRNA expression levels (data not shown) [11]. CD8^+^ T cells from each mouse colony were confirmed to have equivalent levels of CD3, CD8, or Vβ expression (**Figure S1e**). The avidity of the TCR transgenic T cells was assessed by flow cytometry using dilutional tetramer staining as previously described [11], and confirmed to be of similar avidity levels to the clones from which they derived (**Figure S1f**). High avidity and low avidity mice were bred with FVB/N Thy1.2^+^ mice, and pups tested for Vβ and Thy1.2 expression by antibody staining. Avidity status was confirmed by dilutional tetramer staining. Thy1.2 was used as a marker to track adoptively transferred TCR transgenic T cells *in vivo*.

FVB/N-TgN(TIE2GFP)287Sato mice were purchased from Jackson Labs. *Foxp3^gfp^* knock-in mice were provided by Alexander Rudensky (University of Washington, Seattle, WA), and backcrossed to the FVB/N background for 10 generations and then crossed to the *neu*-N background to generate *neu*-N*Foxp3^gfp^* mice [65].

### Cell lines and media

NT2.5, the *neu*-overexpressing mammary tumor cell line, was generated from a spontaneously arising tumor in a *neu*-N mouse as previously described [66], [67]. NT2.5 was maintained in RPMI with 20% FBS, 1.2% HEPES, 1% L-glutamine, 1% non-essential amino acids, 1% sodium pyruvate, 0.5% penicillin-streptomycin, 0.2% insulin, and 0.02% gentamycin. The 3T3neuGM vaccine cell line (3T3 cells genetically modified to express both *neu* and GM-CSF (GM)), and the 3T3GM cell line (mock vaccine that does not express neu) have been previously described [9]. Vaccine lines were maintained in DMEM supplemented with 10% bovine calf serum, 1% sodium pyruvate, and 0.5% penicillin-streptomycin, with methotrexate selection added to the 3T3neuGM line. The T2D^q^ cells [9] were and maintained as previously described in RPMI supplemented with 10% FBS, 1% L-glutamine, 1% sodium pyruvate, 1% non-essential amino acids, 0.5% penicillin-streptomycin, and 0.68% hygromycin B.

### Peptides and antibodies

The RNEU_420–429_ (PDSLRDLSVF) and the negative control peptide LCMV NP_118–126_ (RPQASGVYM) [68] peptides were produced in the Johns Hopkins Biosynthesis and Sequence Facility at a purity >95%. Antibodies used for analysis were anti-Thy1.2-PerCP (Biolegend), anti-TNFα-allophycocyanin (BD Pharmingen), anti-IL-2-FITC (BD Pharmingen), anti-IFNγ-PE (BD Pharmingen), anti-CD4-FITC (BD Pharmingen), anti-CD25-PerCP (Biolegend), anti-CD62L-allophycocyanin (BD Pharmingen), anti-ICOS-PE (anti-CD278, BD Pharmingen), anti-CD44- allophycocyanin (BD Pharmingen), anti-GITR-allophycocyanin (eBioscience), anti-CTLA-4-PE (BD Pharmingen), anti-CD29-PE (eBioscience), anti-LFA-1-PE (anti-CD11a, BD Pharmingen), and anti-CXCR3-allophycocyanin (Biolegend).

### Tumor, vaccine, chemotherapy, and antibody administration procedures

For tumor-clearance studies, FVB/N and *neu*-N mice received subcutaneous (s.c.) injections into the right upper mammary fat pad of 2×10^6^ and 5×10^4^ NT2.5 cells [69], [70] in 0.1 ml PBS, respectively. For tumor infiltrating lymphocyte (TIL) and lymph node analysis experiments, FVB/N and *neu*-N mice received 5×10^6^ and 1×10^6^ NT2.5 cells, respectively. Tumor cells were injected 3 days prior to vaccination, except for TIL and lymph node experiments, where tumor was given 8 days prior to vaccination. Cyclophosphamide (Cy) was injected intraperitoneally (i.p.) at a dose of 100 mg/kg in 0.5 ml PBS. Cy was given 24 hrs prior to vaccine administration. The vaccine (3T3neuGM cells) and mock vaccine (3T3GM cells) were administered s.c. at a dose of 3×10^6^ cells per mouse, divided into three 0.1 ml injections in PBS, and distributed equally among the left upper limb, and right and left lower limb.

### Adoptive transfer studies

Thy1.2^+^ TCR transgenic high avidity and low avidity CD8^+^ T cells were isolated by CD8 negative selection using Dynal CD8 isolation kits (Invitrogen). After isolation, cells were adoptively transferred at 6×10^6^ cells per mouse (unless otherwise stated) in 0.5 ml PBS via tail vein injection, one day after vaccine administration. Four million high avidity T cells was determined to be the minimal adoptive transfer dose necessary to achieve >75% tumor clearance in Cy+ vaccine treated *neu*-N mice (data not shown). A 6×10^6^ T cell dose was administered to maximize cell recovery from various tissues for analysis during cytokine and trafficking experiments after adoptive transfer. T cells were isolated from spleens, lymph nodes, and tumors 3, 5, and 8 days, and 3 and 5 weeks after adoptive transfer and analyzed by flow cytometry as described below.

### Flow cytometry, intracellular cytokine staining (ICS), and tetramer analyses

At the time points described above, spleens and lymph nodes were collected from adoptively transferred mice and mashed through 40 µM nylon cell strainers. Tumors were mashed through cell strainers, enzymatically digested using collagenase (1 mg/ml, Gibco) and Hyluronidase (25 µg/ml, Sigma), and plated on flasks to separate adherent tumor cells from lymphocytes in suspension. Isolated lymphocytes were assessed for activation status by ICS for cytokine expression (IFNγ, IL-2, and TNFα) using a mouse ICS kit (BD Biosciences). Lymphocytes were incubated at 37°C with peptide-pulsed T2D^q^ targets (pulsed with RNEU_420–429_ or NP_118–126_) and Brefeldin A (GolgiStop, BD Biosciences) for 5 hrs using a ratio of lymphocytes to targets of 4∶1. Absolute numbers of CD8^+^ T cells were determined using samples evaluated for CD8 and Thy1.2 expression only. Cells that were stained for cytokine expression were first stained for surface CD8 and Thy1.2 expression. Cells were resuspended in FACS buffer (PBS, 5% FBS, .02% NaAzide) prior to analysis on a FACScalibur (BD Pharmingen). The percentage of antigen-specific activation was calculated by subtracting the percentage of cytokine-secreting cells in NP_118–126_ -pulsed samples from the percentage of cytokine-secreting cells in RNEU_420–429_ -pulsed samples. Absolute numbers were calculated from total T cell counts specific for each peptide. The programs Spice and Pestle were used, courtesy of Mario Roederer of the NIAID Vaccine Research Center, for polycytokine analyses [71].

Treg characterization was assessed using the Foxp3 permeabilization kit (eBioscience) after staining for surface trafficking and activation markers. Surface markers were stained for 2 hours prior to extensive washing, fixation/permeabilization, and Foxp3 staining.

Avidity was assessed by dilutional tetramer analyses using the RNEU_420–429_/H-2D^q^ and NP_118–126_/H-2D^q^ tetramers. Samples were stained using tetramers with a beginning concentration of 1.9 mM and dilutions of 1∶2, 1∶5, 1∶10, 1∶20, 1∶50, 1∶100, 1∶200, 1∶500 (950 nM, 380 nM, 190 nM, 95 nM, 38 nM, 19 nM, 9.5 nM, and 3.8 nM respectively) was used to assess T cell avidity by flow cytometry [11].

### Tumor size assessments

Tumors were measured twice weekly, beginning 3 days after vaccination. Tumor length and width were measured and used to calculate tumor area. Mice with tumors >1 cm^2^ were sacrificed according to Johns Hopkins Animal Care and Use protocols.

### Biodistribution studies and *in vivo* imaging

At various time points after adoptive transfer, mice were injected i.v. with 20 µg of Thy1.2 monoclonal antibody or control IgG radiolabeled with 200 µCi indium-111 per mouse. The antibody was radiolabeled using previously described methods [12], [72], [73]. After 24 hours, lymph nodes, spleens, and tumors were excised, weighed, and placed in a gamma counter to measure their radioactivity. For *in vivo* imaging, mice were treated as described above. Twenty-four hours after radiolabeled antibody injection the mice were anesthetized with 3% isofluorane and maintained at 1–2% with a constant rate of oxygen flow of 1 l/m. Mice were then imaged by small-animal SPECT/CT (XSPECT system, by GammaMedica) fitted with a pinhole collimator appropriate for indium-111 photons.

### *In vitro* suppression assay

CD11c^+^ cells were isolated from the vaccine-draining lymph nodes (VDNs) of FVB/N mice that had been vaccinated 7 days prior to dissection. Lymph nodes were gently mashed through 40 µM cell strainers and then CD11c^+^ cells were positively selected using CD11c^+^ MicroBeads (Miltenyi Biotec). Tregs were isolated from the spleens and lymph nodes of *Foxp3^GFP^ neu*-N mice that had been vaccinated 7 days prior to isolation. CD4^+^ T cells were isolated using negative selection Dynal CD4^+^ T cell isolation kits (Invitrogen) and then stained for CD25 expression using anti-CD25-PE (eBioscience) prior to sorting. Foxp3 expression was indicated by GFP fluorescence and T cells were sorted into either GFP^+^CD25^high^ or GFP^+^CD25^low^ populations. Control CD4^+^ T cells were isolated from non-vaccinated FVB/N GFP mice using CD4^+^ negative selection. High avidity T cells were isolated from TCR transgenic mice using CD8^+^ T cell negative selection Dynal kits (Invitrogen). High avidity T cells were then labeled with CFSE using the CellTrace CFSE Proliferation Kit (Invitrogen). CDllc^+^ dendritic cells (DC), Tregs/CD4^+^ T cells, and high avidity T cells were plated at a 1∶1∶2 ratio. Unstimulated high avidity T cells were plated as a negative control. CD11c^+^ cells were plated with high avidity cells in a 1∶2 ratio and FVB/N-derived CD4^+^ T cells, CD11c^+^ cells, and high avidity cells plated at a 1∶2∶1 ratio, both as positive controls. Cells were incubated for 3 and 5 days prior to flow cytometric analysis of CFSE dilution on Thy1.2^+^ cells.

### *In vivo* suppression assay

*Neu*-N/*Foxp3^gfp^* mice were injected with 1×10^5^ NT2.5 cells followed by whole cell vaccination 3 days later. Seven days post-vaccination, splenic CD4^+^ T cells were isolated by negative selection (Invitrogen), and sorted based on FoxP3 and CD25 (Clone 3C7, Biolegend) expression. CD4^+^FoxP3^+^CD25 high or low cells (1×10^6^) were adoptively transferred into FVB/N-TgN(TIE2GFP)287Sato mice that were vaccinated 1 day later. Two weeks post-vaccination CD8^+^ T cells were negatively isolated from the FVB/N-TgN(TIE2GFP)287Sato mice and tested for IFN-γ secretion by ICS assay, and the %IFN-γ^+^ CD8^+^ T cells was evaluated.

### Immunohistochemistry (IHC)

One week after receiving a neu-expressing tumor cells, *neu*-N mice were treated with Cy, vaccine, and adoptively transferred high avidity T cells. On day 3 or 5 after treatment, tumors from *neu*-N mice were fixed in 10% paraformaldehyde on days 3 and 5 after adoptive transfer. Tumors were imbedded in paraffin and sectioned by the Johns Hopkins Medical Laboratories, Reference Histology Lab. Slides were then stained in the Johns Hopkins IHC laboratory. The anti-mouse CXCL9 antibody (R & D Systems) was used at a 1∶200 dilution with an anti-goat antibody used at a 1∶2000 dilution. The Leica-Bond maX system with Bond-max Software was used, and the epitope retrieval method used was Leica-Bond ER2.

For immunofluorescence staining of Thy1.2 in the tumors of treated mice, tumors were frozen in Tissue-Tek OCT (VWR) prior to preparation of 5 µm sections by the JHMI Pathology core. Anti-Thy1.2-biotin (BD Pharmingen) and AlexaFluor 488 streptavidin (Invitrogen) were used with standard immunofluorescence protocols. Blocking was completed with an avidin and biotin blocking kit (Vector labs). Slides were then mounted using ProLong Gold anti-fade reagent plus DAPI stain (Molecular Probes).

### Statistical analysis

Statistical analyses were conducted in Microsoft Excel using either ANOVA analysis or an unpaired, two-tailed, Student's t-test, assuming equal population variances. A log-rank test was used for Kaplan Meier plots.

## Supporting Information

Figure S1 **Characterization of RNEU_420–429_-specific, CD8^+^ TCR transgenic mice.** Staining CD8^+^ T cells from high and low avidity mice confirms the dominance of the transgenes. (**A**) CD8^+^ T cells from high avidity mice are >98% Vβ4^+^. CD8^+^ T cells from an FVB/N control are ∼10% Vβ4^+^. CD8^+^ T cells from low avidity mice stain >95% Vβ2^+^. CD8^+^ T cells from an FVB/N control are ∼17% Vβ2^+^. (**B**) Dilutional tetramer staining confirms avidity of TCR transgenic CD8^+^ T cells. FVB/N mice stain similarly with RNEU_420–429_/H-2D^q^ tetramer (green line) and irrelevant peptide NP_118–126_/H-2D^q^ tetramer (blue line) at 1∶5, 1∶50, and 1∶200 dilutions. Low avidity TCR transgenic mice show a positive shift in RNEU_420–429_/H-2D^q^ tetramer staining at 1∶5 and 1∶50 dilutions when compared to NP_118–126_/H-2D^q^ tetramer staining. High avidity TCR transgenic mice show a strong positive shift of RNEU_420–429_/H-2D^q^ tetramer staining at all dilutions when compared to NP_118–126_/H-2D^q^ tetramer staining. (**C**) Comparison of CD8, CD3, and Vβ expression on T cells from TCR transgenic mice using antibody staining. There is no difference in expression of these markers across mouse lines.
(TIF)

Figure S2 **High and low avidity T cells persist longer in FVB/N than in** ***neu*** **-N mice after adoptive transfer.** The average percent of Thy1.2^+^ high or low avidity T cells of the total CD8^+^ T cells in FVB/N or *neu*-N mice as a measure of cell persistence at 1, 3, and 5 weeks post-adoptive transfer. **Treatments:** Tumor Alone = tumor plus adoptive transfer, Mock = tumor, 3T3GM mock vaccine, and adoptive transfer, Vacc = tumor, 3T3neuGM vaccine, and adoptive transfer. Cy Mock = tumor, Cy, 3T3GM mock vaccine, and adoptive transfer, Cy Vacc = tumor, Cy, 3T3neuGM vaccine, and adoptive transfer. High avidity T cell transfer = gray bars; low avidity T cell = white bars. (**A**) Adoptive transfer of high or low avidity T cells into treated FVB/N mice. (**B**) Adoptive transfer of high or low avidity T cells into treated *neu*-N mice. Note different scale on the Y axis. ** = p<.0001.
(TIF)

Figure S3 **Polycytokine secreting high and low avidity T cells are detected following adoptive transfer into treated FVB/N mice.** Lymphocytes were collected from tumor-draining lymph nodes on day 3 and day 5 and analyzed for cytokine secretion by ICS as described in the methods. The absolute # of activated Thy1.2 cells in the tumor-draining nodes (TDN, Red Bars), vaccine-draining nodes (VDN, Green Bars), and non-draining nodes (NDN, Blue Bars) on Day 3, 5, and 8 after adoptive transfer that produce various combinations of IFNγ, TNFα, and IL-2, is shown. (n = 3 mice per group). These experiments were repeated at least three times with similar results. (**A**) High avidity T cell transfer with 3T3GM Mock (Left) or 3T3neuGM vaccine (Right). (**B**) High avidity T cell transfer with Cy plus 3T3GM Mock (Left) or 3T3neuGM vaccine (Right). (**C**) Low avidity T cell transfer with Cy plus 3T3GM Mock (Left) or 3T3neuGM vaccine (Right). Polyclonal cytokine expression was not detected at high levels in low avidity T cells transferred into FVB/N mice treated with vaccine or mock vaccine without Cy (data not shown).
(TIF)

Figure S4 ***Neu*** **-N mouse tumors produce higher levels of CXCL9 following treatment with Cy and adoptively transferred high avidity T cells.** CXCL9 staining of neu-expressing tumors on day 3 (Left Panels) or 5 (Right Panels) after treatment as described in the Methods, Top panels: High avidity T cell transfer+3T3neuGM vaccine. Middle panels: Cy+3T3neuGM vaccine. Lower panels: High avidity T cell transfer+Cy+3T3neuGM vaccine.
(TIF)
