# Supplementary figures and images for: Trafficking of High Avidity HER-2/neu-Specific T Cells into HER-2/neu-Expressing Tumors after Depletion of Effector/Memory-Like Regulatory T Cells
Source: PLoS One. 2012 Feb 16;7(2):e31962. doi: 10.1371/journal.pone.0031962 (PMC3281086; doi:10.1371/journal.pone.0031962)

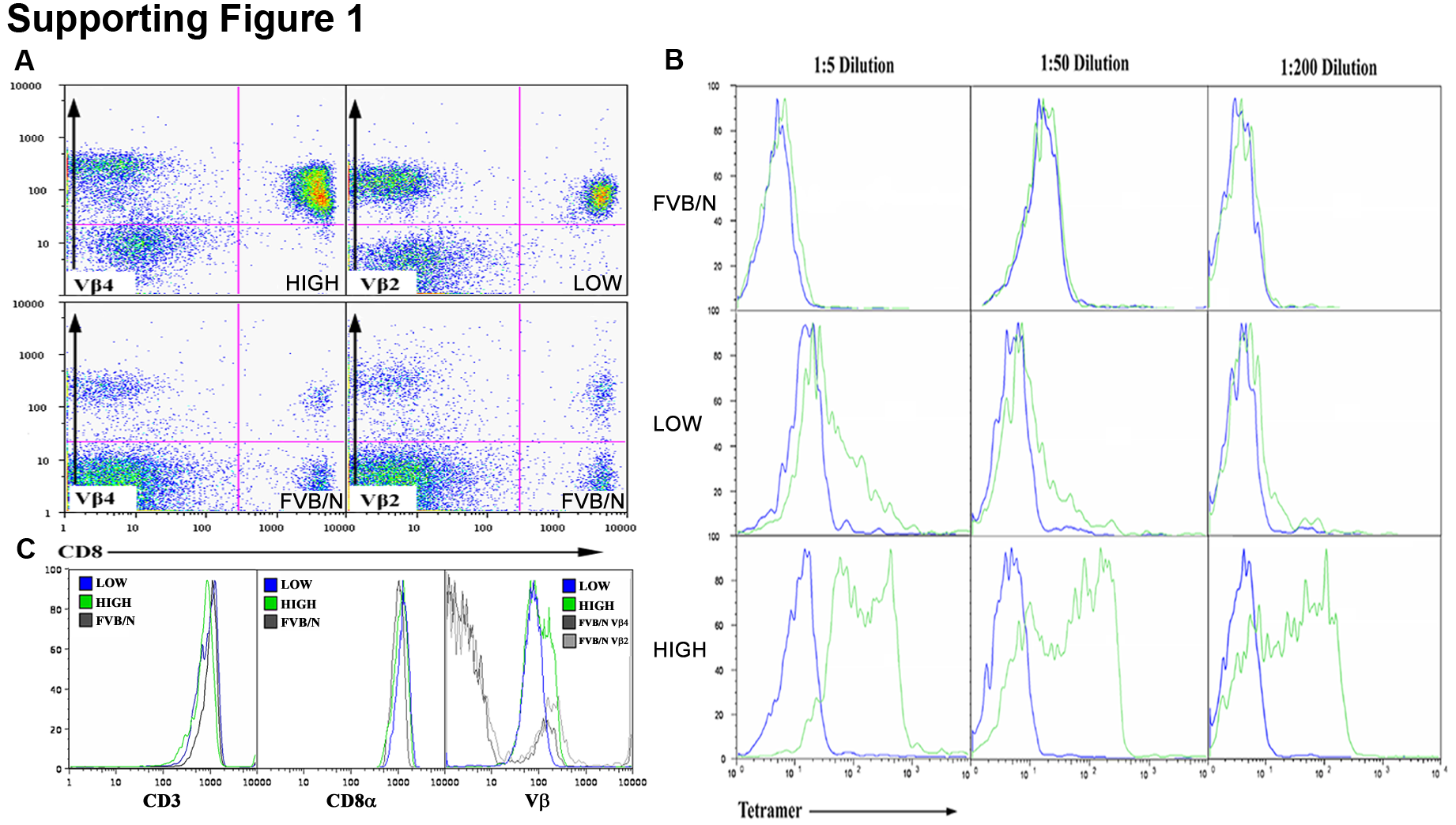

Supplement: Figure S1 — Characterization of RNEU420–429-specific, CD8+ TCR transgenic mice. Staining CD8+ T cells from high and low avidity mice confirms the dominance of the transgenes. (A) CD8+ T cells from high avidity mice are >98% Vβ4+. CD8+ T cells from an FVB/N control are ∼10% Vβ4+. CD8+ T cells from low avidity mice stain >95% Vβ2+. CD8+ T cells from an FVB/N control are ∼17% Vβ2+. (B) Dilutional tetramer staining confirms avidity of TCR transgenic CD8+ T cells. FVB/N mice stain similarly with RNEU420–429/H-2Dq tetramer (green line) and irrelevant peptide NP118–126/H-2Dq tetramer (blue line) at 1∶5, 1∶50, and 1∶200 dilutions. Low avidity TCR transgenic mice show a positive shift in RNEU420–429/H-2Dq tetramer staining at 1∶5 and 1∶50 dilutions when compared to NP118–126/H-2Dq tetramer staining. High avidity TCR transgenic mice show a strong positive shift of RNEU420–429/H-2Dq tetramer staining at all dilutions when compared to NP118–126/H-2Dq tetramer staining. (C) Comparison of CD8, CD3, and Vβ expression on T cells from TCR transgenic mice using antibody staining. There is no difference in expression of these markers across mouse lines. (TIF) [file pone.0031962.s001.tif]

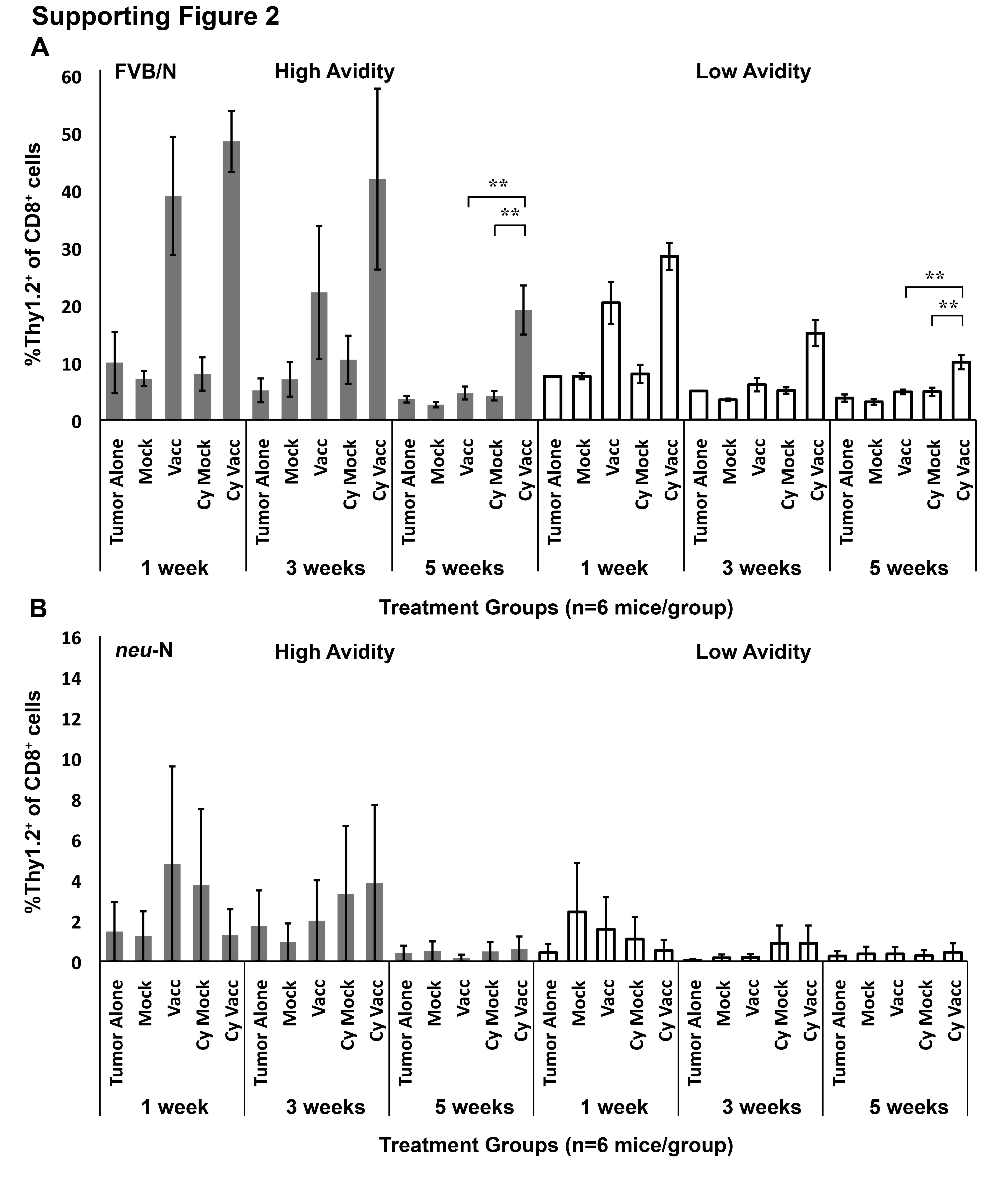

Supplement: Figure S2 — High and low avidity T cells persist longer in FVB/N than in neu -N mice after adoptive transfer. The average percent of Thy1.2+ high or low avidity T cells of the total CD8+ T cells in FVB/N or neu-N mice as a measure of cell persistence at 1, 3, and 5 weeks post-adoptive transfer. Treatments: Tumor Alone = tumor plus adoptive transfer, Mock = tumor, 3T3GM mock vaccine, and adoptive transfer, Vacc = tumor, 3T3neuGM vaccine, and adoptive transfer. Cy Mock = tumor, Cy, 3T3GM mock vaccine, and adoptive transfer, Cy Vacc = tumor, Cy, 3T3neuGM vaccine, and adoptive transfer. High avidity T cell transfer = gray bars; low avidity T cell = white bars. (A) Adoptive transfer of high or low avidity T cells into treated FVB/N mice. (B) Adoptive transfer of high or low avidity T cells into treated neu-N mice. Note different scale on the Y axis. ** = p<.0001. (TIF) [file pone.0031962.s002.tif]

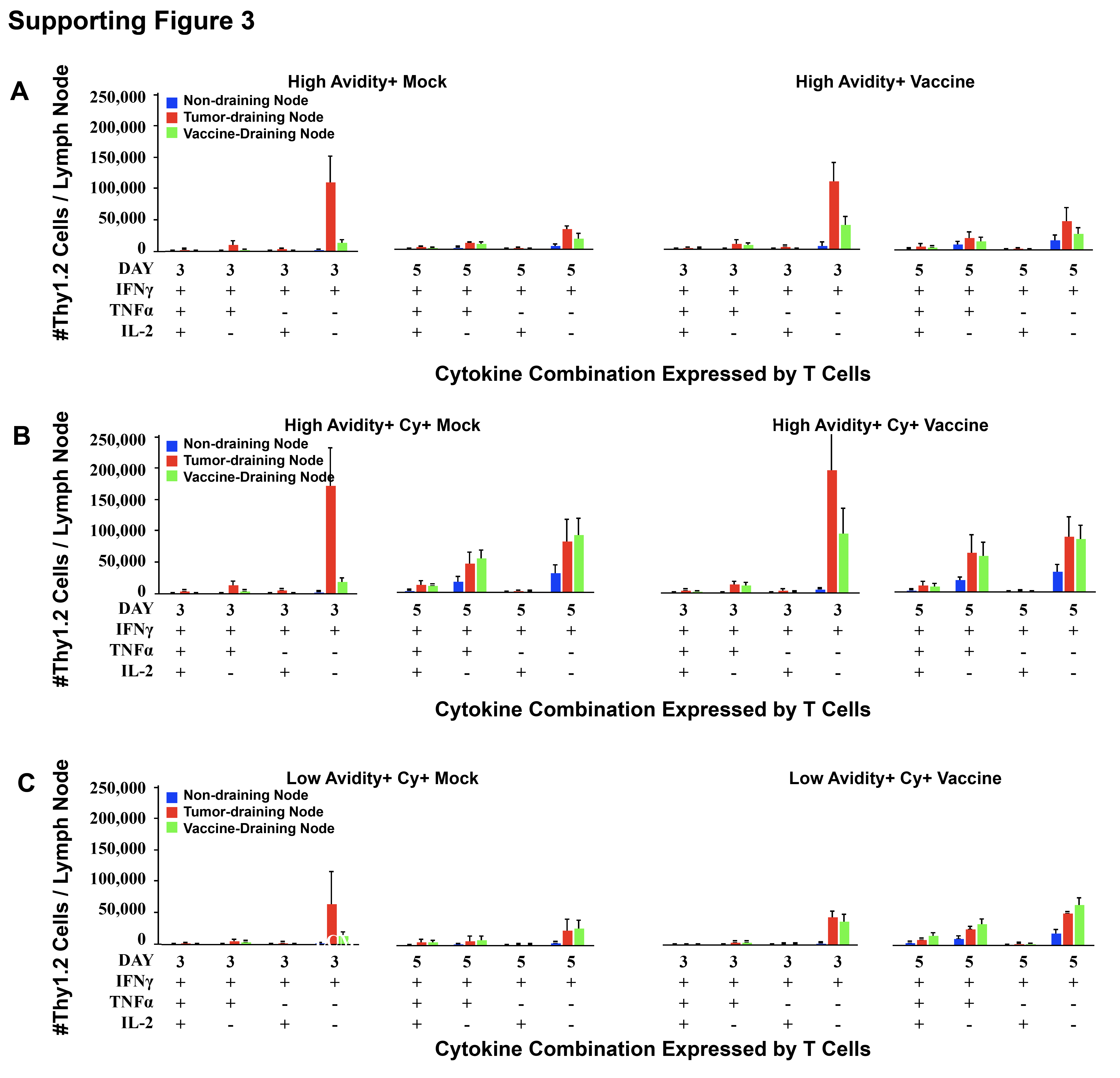

Supplement: Figure S3 — Polycytokine secreting high and low avidity T cells are detected following adoptive transfer into treated FVB/N mice. Lymphocytes were collected from tumor-draining lymph nodes on day 3 and day 5 and analyzed for cytokine secretion by ICS as described in the methods. The absolute # of activated Thy1.2 cells in the tumor-draining nodes (TDN, Red Bars), vaccine-draining nodes (VDN, Green Bars), and non-draining nodes (NDN, Blue Bars) on Day 3, 5, and 8 after adoptive transfer that produce various combinations of IFNγ, TNFα, and IL-2, is shown. (n = 3 mice per group). These experiments were repeated at least three times with similar results. (A) High avidity T cell transfer with 3T3GM Mock (Left) or 3T3neuGM vaccine (Right). (B) High avidity T cell transfer with Cy plus 3T3GM Mock (Left) or 3T3neuGM vaccine (Right). (C) Low avidity T cell transfer with Cy plus 3T3GM Mock (Left) or 3T3neuGM vaccine (Right). Polyclonal cytokine expression was not detected at high levels in low avidity T cells transferred into FVB/N mice treated with vaccine or mock vaccine without Cy (data not shown). (TIF) [file pone.0031962.s003.tif]

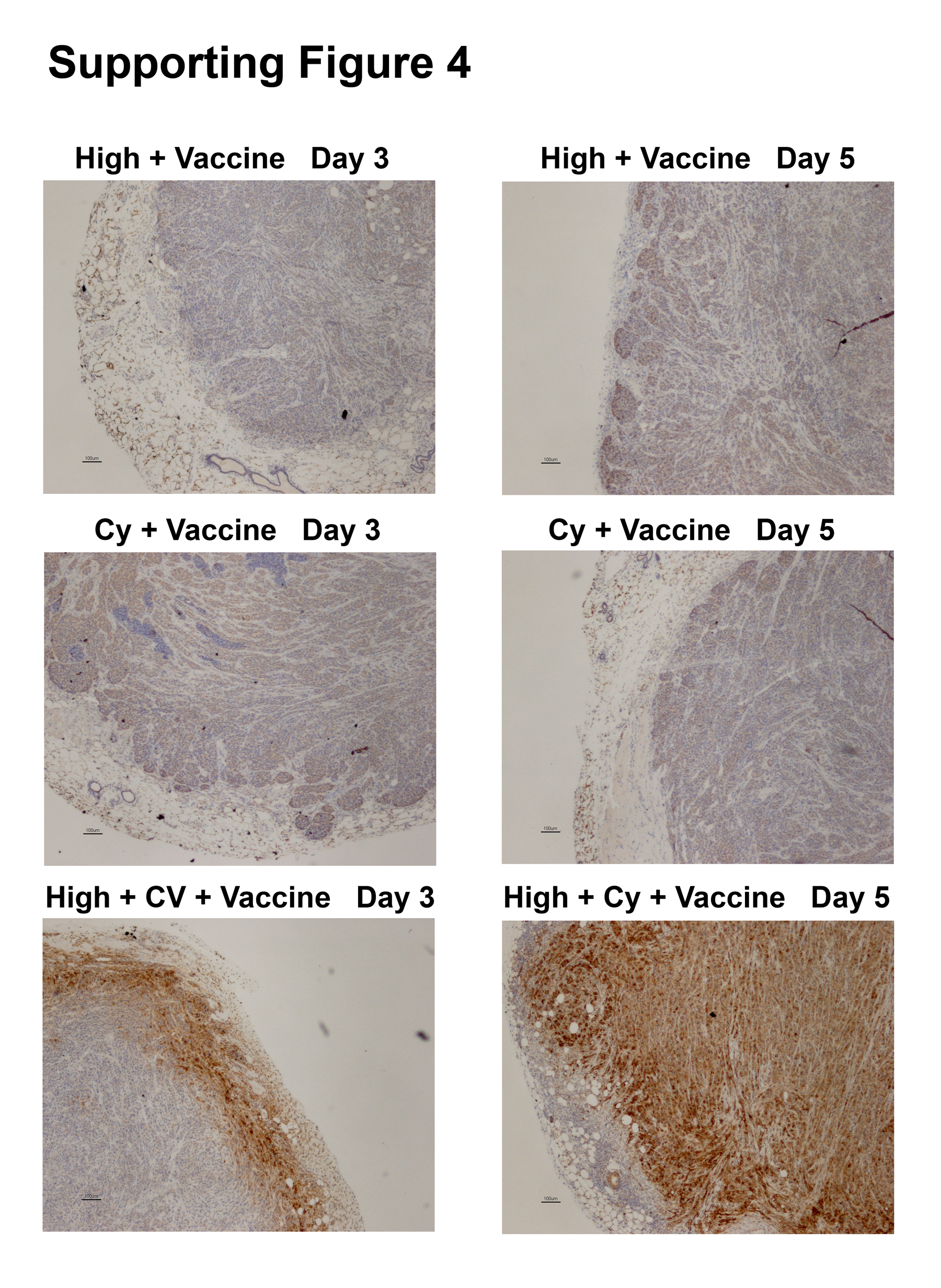

Supplement: Figure S4 — Neu -N mouse tumors produce higher levels of CXCL9 following treatment with Cy and adoptively transferred high avidity T cells. CXCL9 staining of neu-expressing tumors on day 3 (Left Panels) or 5 (Right Panels) after treatment as described in the Methods, Top panels: High avidity T cell transfer+3T3neuGM vaccine. Middle panels: Cy+3T3neuGM vaccine. Lower panels: High avidity T cell transfer+Cy+3T3neuGM vaccine. (TIF) [file pone.0031962.s004.tif]
